# Localized delivery and retention of hydrogen sulfide causing regional lipid accumulation in mouse adipose tissues in vivo

**DOI:** 10.1038/s42003-025-08353-9

**Published:** 2025-07-01

**Authors:** Richa Verma, Ming Fu, Hassan M. Arif, Guangdong Yang, Ishani Sarkar, Kuljeet Kaur, John B. Matson, Lingyun Wu, Rui Wang

**Affiliations:** 1 Cardiovascular and Metabolic Research Unit, Laurentian University, Sudbury, ON Canada; 2 College of Basic Medicine, Shandong Second Medical University, Weifang, China; 3 Department of Biology, York University, Toronto, ON Canada; 4 Department of Chemistry, Macromolecules Innovation Institute, and Virginia Tech Centre for Drug Discovery, Blacksburg, VA USA

**Keywords:** Mechanisms of disease, Cellular imaging, Lipids, Biological techniques

## Abstract

Therapeutical application of hydrogen sulfide (H_2_S) is limited due to the lack of delivery routes for specific organs and the rapid and wide dispersal of H_2_S in vivo. While H_2_S shows adipogenic effects in vitro, its in vivo impacts on obesity remain unclear. This study applies a H_2_S-slow-releasing hydrogel (H_2_S gel) to deliver H_2_S locally in subcutaneous adipose tissue and examines local lipid accumulation in mice. H_2_S is released from H_2_S gels within 6 h and lasts for 72 h, elevating H_2_S levels in local adipose tissue but not in the plasma. Localized H_2_S gel delivery causes significant lipid accumulation and larger lipid droplet diameter in mouse adipose tissues. The expressions of sterol regulatory element-binding protein, peroxisome proliferator-activated receptor-γ, adiponectin, and perilipin are all upregulated by H_2_S gel injections. Local delivery and retention of H_2_S in adipose tissues increase lipid accumulation more in wild-type than in cystathionine-γ-lyase knockout mice. This study confirms the feasibility of selectively delivering H_2_S via injectable hydrogels and their effectiveness in regulating targeted tissue functions. Furthermore, this work deepens our understanding of the role of H_2_S in obesity development under physiological conditions and offers a practical implementation strategy for H_2_S-based therapeutic interventions.

## Abstract (web summary)

Locally delivered H_2_S slow-releasing hydrogel promotes lipid accumulation, adipocyte hypertrophy, macrophage infiltration, and fibrosis in mouse adipose tissue, offering a practical implementation strategy for H_2_S-based interventions.

## Introduction

Obesity is one of the most common morbidities, characterized by excessive body fat accumulation that adversely affects health. It is manifested with an overabundance of malfunctioning adipose tissues due to genetic and epigenetic factors as well as unhealthy lifestyles^1,2^. It can also lead to other disorders, including dyslipidemia, diabetes, cardiovascular diseases, osteoarthritis, neurodegenerative diseases, and certain types of cancer^3–5^. Adipocytes or fat cells play a crucial role in regulating energy metabolism and maintaining healthy body weight. The number of these cells are tightly controlled, and any disruption to this regulation can lead to obesity and other metabolic disorders^6^. Adipocyte hypertrophy (an increase in size) or hyperplasia (an increase in quantity) also contribute to the development of obesity. During periods of excess calorie intake, adipocytes tend to grow in size and produce adipokines, which attract additional pre-adipocytes and stimulate their maturation into adipocytes. When the capacity to recruit and expand adipocytes is exceeded, lipid accumulates in areas such as visceral depots, the liver, and skeletal muscle. These changes can lead to inflammation, insulin resistance, and other symptoms of metabolic syndrome^6–8^.

H_2_S contributes to the homeostatic regulation of the cardiovascular, pulmonary, gastroenterological, neurological, immunological, and endocrine systems^9–11^. Previously, we showed that exogenous H_2_S increased adipogenesis and lipid accumulation in mouse adipocytes^12–14^. Cystathionine γ-lyase (CSE) is the major enzyme involved in the production of H_2_S in adipose tissue. Upon knocking out the CSE gene (KO) in mice, we observed a significant decrease in H_2_S production in primary mouse adipocytes^14^. When CSE-KO preadipocytes were stimulated to differentiation, they showed a significantly lower adipogenesis and lipid accumulation than WT preadipocytes. Furthermore, the addition of exogenous H_2_S to the culture media increased adipogenesis and lipid accumulation in adipocytes in vitro^12^. However, in vitro cellular and molecular studies can never fully replicate in vivo physiological conditions. The physiological effects of H_2_S in vivo would be significantly impacted by the metabolism of this gasotransmitter, including its tissue/organ-specific production, absorption, distribution, and excretion. Interactions of different types of cells and tissues in the presence of circulation and numerous endogenous factors would determine the net effect of H_2_S on its targets in vivo. It is thus merited to investigate whether an elevated H_2_S level under in vivo conditions can induce adipogenesis and lipogenesis, one critical evidence for establishing a pro-obesity role of H_2_S.

**Fig. 1:**
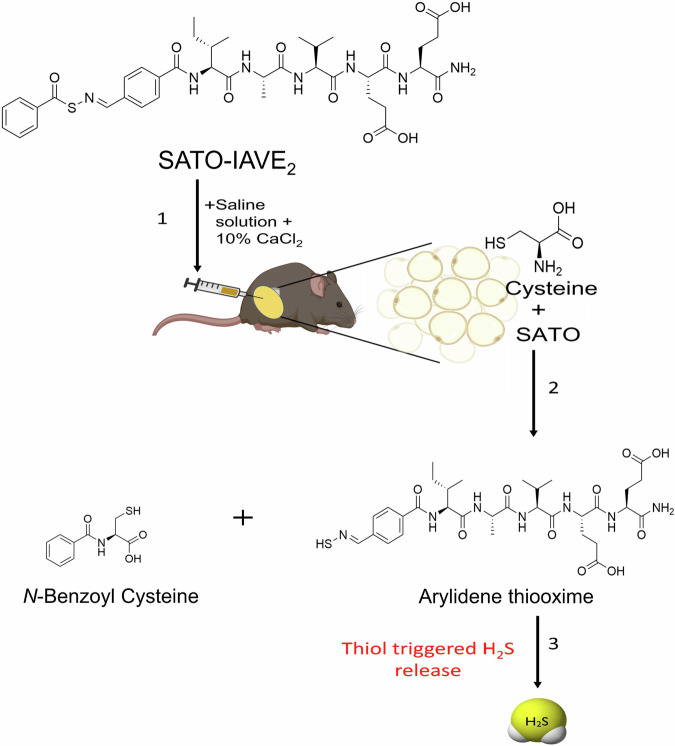
Mechanism for H_2_S release from H_2_S releasing gel (SATO-IAVE_2_). H_2_S gel in the presence of CaCl_2_, forms a biodegradable hydrogel (1). The SATO group, upon contact with thiols such as cysteine, undergoes chemical transformations, forming arylidenethiooxime and N-benzoylated cysteine (2). Arylidenethiooxime decomposes, releasing H_2_S (3). Created with BioRender. Verma, R. (2025) https://BioRender.com/dbuw3sa.

The physiological effects of endogenous H_2_S can be mimicked by exogenously applied H_2_S salts and donors like NaHS and GYY4137^15,16^. While selective over-expression of H_2_S-generating enzymes in regional adipose tissue in vivo is a daunting challenge, systemic administration of H_2_S-delivering compounds would generate system-wide alteration of H_2_S levels, which cannot fulfill the mission to produce regionalized delivery and retention of exogenous H_2_S for the targeted tissues or organs. H_2_S gel is a thiol-triggered H_2_S-releasing aromatic peptide amphiphile hydrogel^17^ (Fig. 1). In the present study, we utilized H_2_S gel as the means for directly and restrictively delivering H_2_S to subcutaneous adipose tissues in mice. We investigated whether this administration approach yielded the desired target-specific H_2_S release and retention. The development of adipogenesis and lipogenesis in the injected region in vivo was subsequently examined.

## Results

### In vitro assessment of H_2_S release kinetics and gel degradation of H_2_S gel

**Fig. 2:**
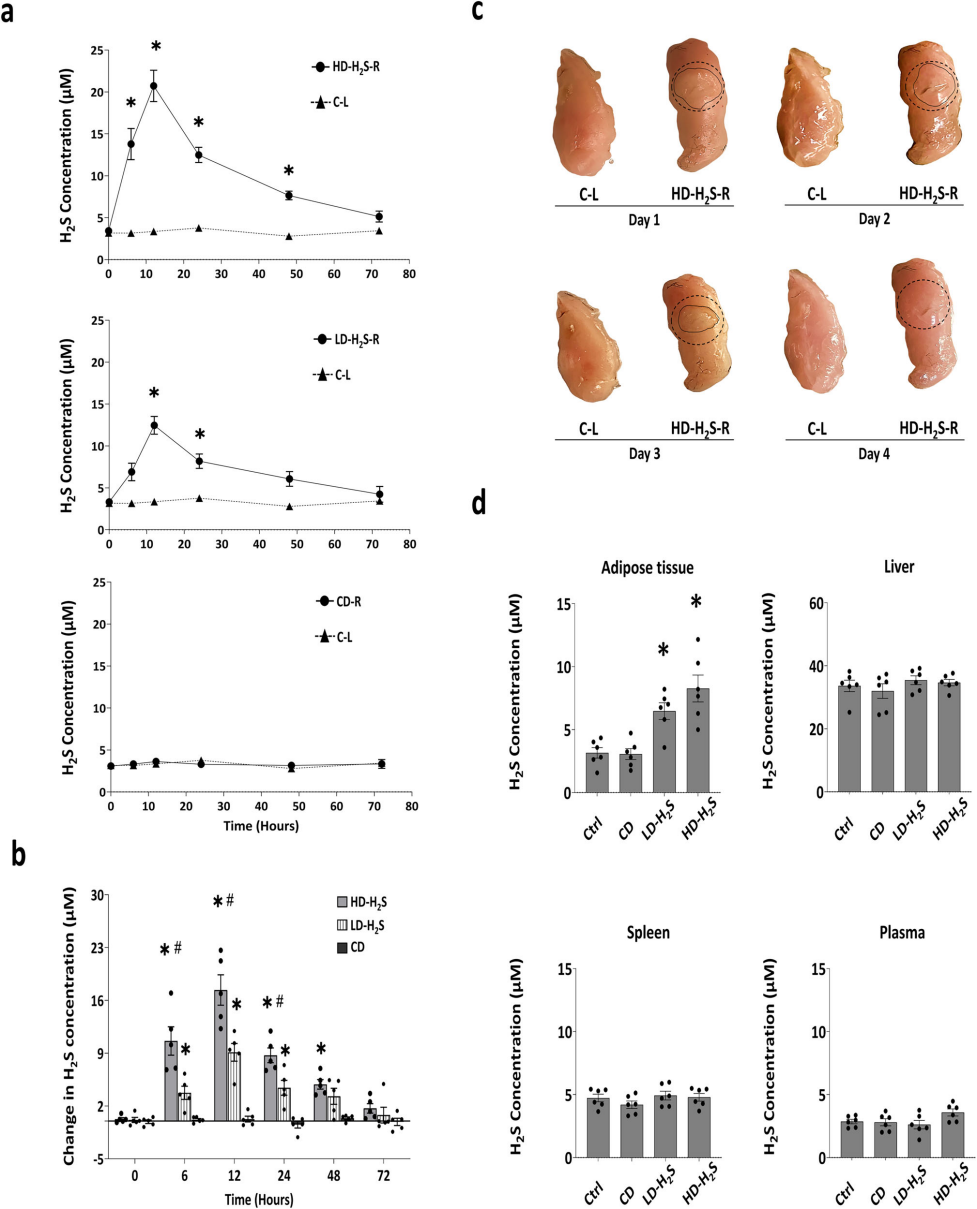
H_2_S release from H_2_S gel and its degradation in vitro. **a** In vitro H_2_S release study of three peptide amphiphiles—HD-H_2_S, LD-H_2_S, CD compared to control samples (C-L) without injections, assessed via HPLC. *n* = 5 independent replicates for each time point. *p < 0.05 vs C-L. **b** Net H_2_S released from peptide gels by subtracting H_2_S levels in C-L tissues from those in HD-H_2_S, LD-H_2_S, CD-injected adipose tissues. *p < 0.05 vs. CD, and #*p* < 0.05 vs. LD-H_2_S. **c** Morphological changes of WT adipose tissues injected with or without HD-H_2_S. Dashed circles mark the original area with HD-H_2_S gel injection, while solid-line contours with the pale coloration indicate the actual visualization of the injected HD-H_2_S gel. **d** H_2_S levels in adipose tissues and plasma of WT mice receiving HD-H_2_S, LD-H_2_S, and CD injections for 30 days or no injection (Ctrl). *n* = 6 independent mouse tissue replicates. Statistical significance is shown as *p < 0.05 vs. Ctrl and CD. Abbreviations: HD-H_2_S-R: High dose (78 µmol/kg) H_2_S-releasing gel injected in right flank adipose tissue; LD-H_2_S-R: Low dose (39 µmol/kg) H_2_S-releasing gel; CD-R: Control gel (non-H_2_S-releasing) injected in right flank; C-L: Uninjected left flank adipose tissue control used as an internal control for each mouse; Ctrl: mouse given no injection. The error bars in this figure represent standard error.

Twelve hours after H_2_S gel injection into the collected WT mouse adipose tissue lysate in vitro, both HD-H_2_S and LD-H_2_S gel released H_2_S to the maximal level, and it lasted more than 48 h (Fig. 2a). CD injections did not result in H_2_S release. The maximum amounts of H_2_S released from HD-H_2_S and LD-H_2_S were 21 ± 2 µM/mg and 12 ± 1 µM/mg, respectively (Fig. 2b). On Day 4, the HD-H_2_S injected pale-coloured area disappeared, indicating complete degradation of the injected HD-H_2_S (Fig. 2c).

### H_2_S concentration in H_2_S gel-injected WT adipose tissue and surrounding tissues

After 30 days of H_2_S gel injections in vivo, WT mouse adipose tissue injected with LD-H_2_S and HD-H_2_S showed a local H_2_S concentration of 8 ± 3 µM/mg, and 6 ± 2 µM/mg significantly higher than those of non-injected (Ctrl) and CD-injected adipose tissues. H_2_S concentrations in the liver, spleen, and plasma of WT mice did not show any significant difference among the tested groups (Fig. 2d).

### H_2_S levels and CSE expression in H_2_S gel-injected WT adipose tissue in vivo

**Fig. 3:**
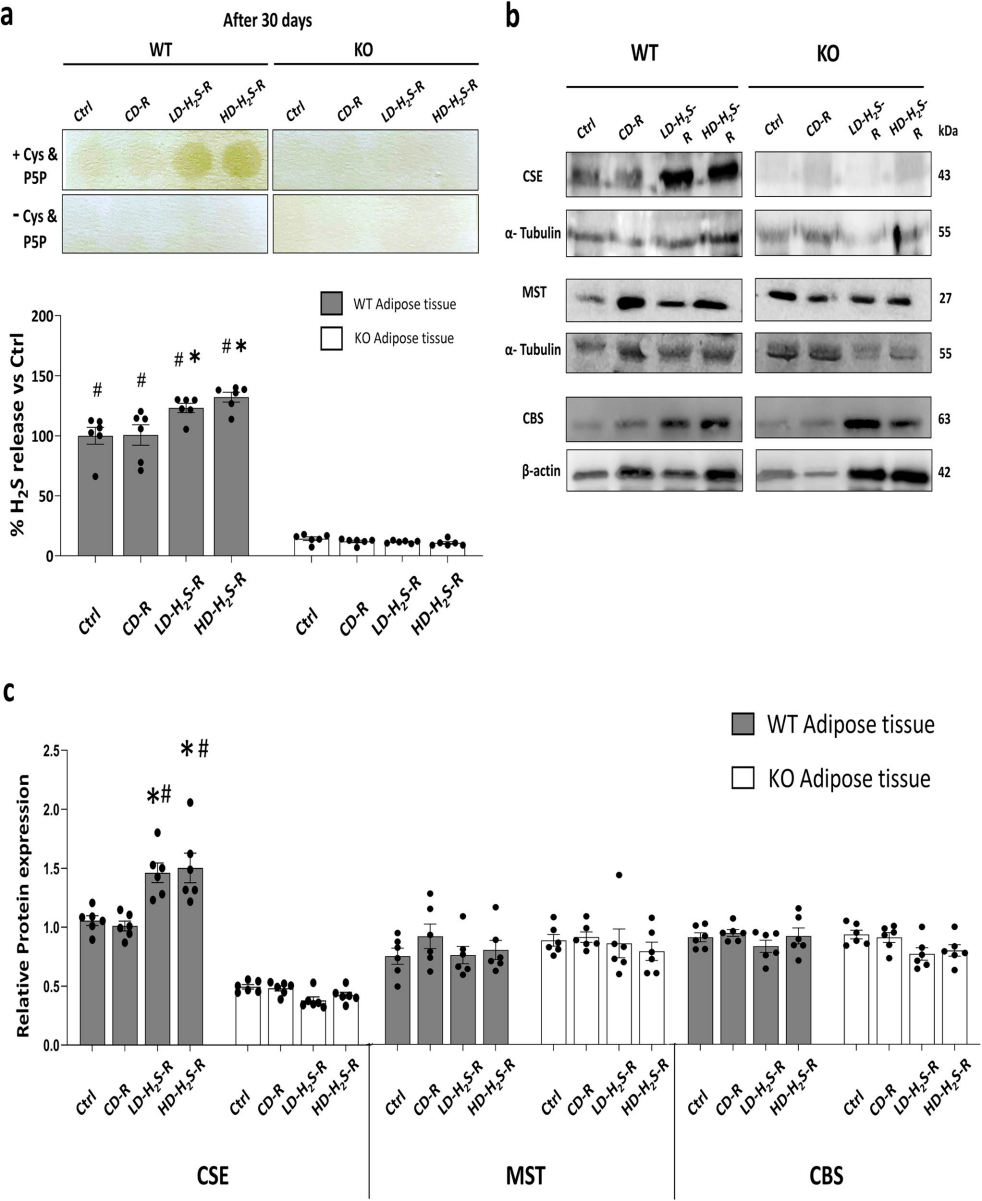
H_2_S gel increased H_2_S levels and CSE expression in WT adipose tissue. **a** Changes in H_2_S levels in adipose tissues of wild-type (WT) and CSE-knockout (KO) mice after 30 days of injection with HD-H_2_S, LD-H_2_S, and CD preparations relative to baseline H_2_S levels in untreated WT mouse adipose tissues (Ctrl). *n* = 6 mouse replicates. *p < 0.05 vs. Ctrl and CD, and #*p* < 0.05 vs. CSE-KO mouse tissues. **b** Expression levels of CSE, CBS, and MST proteins in WT and CSE-KO mouse adipose tissues after 30 days of treatments. **c** Relative expressions of CSE, CBS, and MST to β actin and α tubulin. *n* = 6; *p < 0.05 vs. Ctrl and CD, #*p* < 0.05 vs. KO mouse tissues. Cys stands for Cysteine; P5P stands for Pyridoxial 5′-phosphate. The error bars in this figure represent standard error.

For comparing the changes in total H_2_S levels in the tissue after 30 days of study, we utilized the lead acetate method. WT adipose tissues exhibited significantly higher H_2_S levels than that of CSE-KO tissues, as shown by the intensity of darkening on the lead acetate papers. Untreated WT adipose tissues exhibited a 56% higher H_2_S level than that of untreated CSE-KO adipose tissues. H_2_S gel injections increased H_2_S levels in WT adipose tissues, but not in CSE-KO, in vivo (Fig. 3a). CSE, CBS, and MST proteins were all expressed in WT adipose tissues. HD-H_2_S and LD-H_2_S treatments upregulated CSE expression in WT adipose tissues compared with the tissues that received non-injection (Ctrl) or CD. MST protein was abundant, but CSE protein was completely undetectable in CSE-KO mice adipose tissues (Fig. 3b, c). There were no significant changes in MST or CBS expression in any group following H_2_S gel injections, and the expression levels of CSE, MST, and CBS proteins in the adipose tissue of H_2_S gel-injected CSE-KO mice remained unaltered (Fig. 3b, c).

### The effects of H_2_S gel on lipid accumulation in WT adipose tissue in vivo

**Fig. 4:**
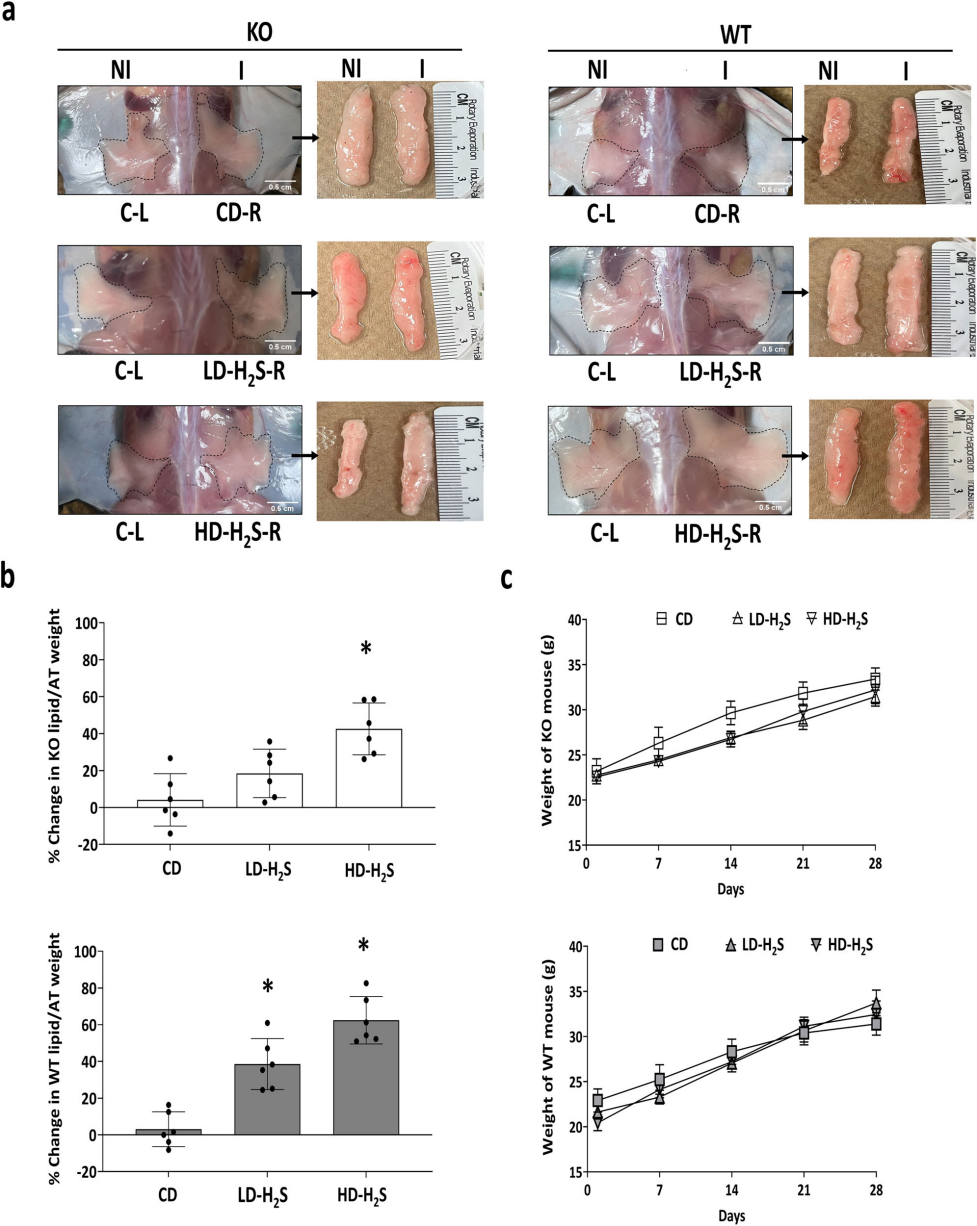
H_2_S gel increased lipid content in WT and CSE-KO mouse adipose tissues. **a** Lipid accumulation in CSE-KO and WT subcutaneous adipose tissues outlined by dotted contours. **b** Changes in lipid content/adipose tissue weight in injected vs. non-injected controls for CSE KO and WT mice. **c** Changes in mouse body weights of CSE KO and WT mice. *n* = 6 mouse replicates. *p < 0.05 vs. % change in CD. I stands for Injected side; NI stands for Non-injected side of the same mice; AT stands for Adipose tissue. The error bars in this figure represent standard error.

After 30 days of local injections of H_2_S gel, significant increases in lipid content were observed in CSE-KO adipose tissue injected with HD-H_2_S, but not with LD-H_2_S (Fig. 4a, b). In both WT adipose tissues, HD-H_2_S and LD-H_2_S induced significantly higher lipid accumulation (Fig. 4a, b). Mice injected with CD showed no change in adipose tissue weight or lipid content in the injected adipose tissue of CSE-KO and WT mice (Fig. 4a, b). No change in mouse overall body weights was observed in any animal groups (Fig. 4c).

### The effects of H_2_S gel on the expressions of adipogenic and lipogenic marker proteins in vivo

**Fig. 5:**
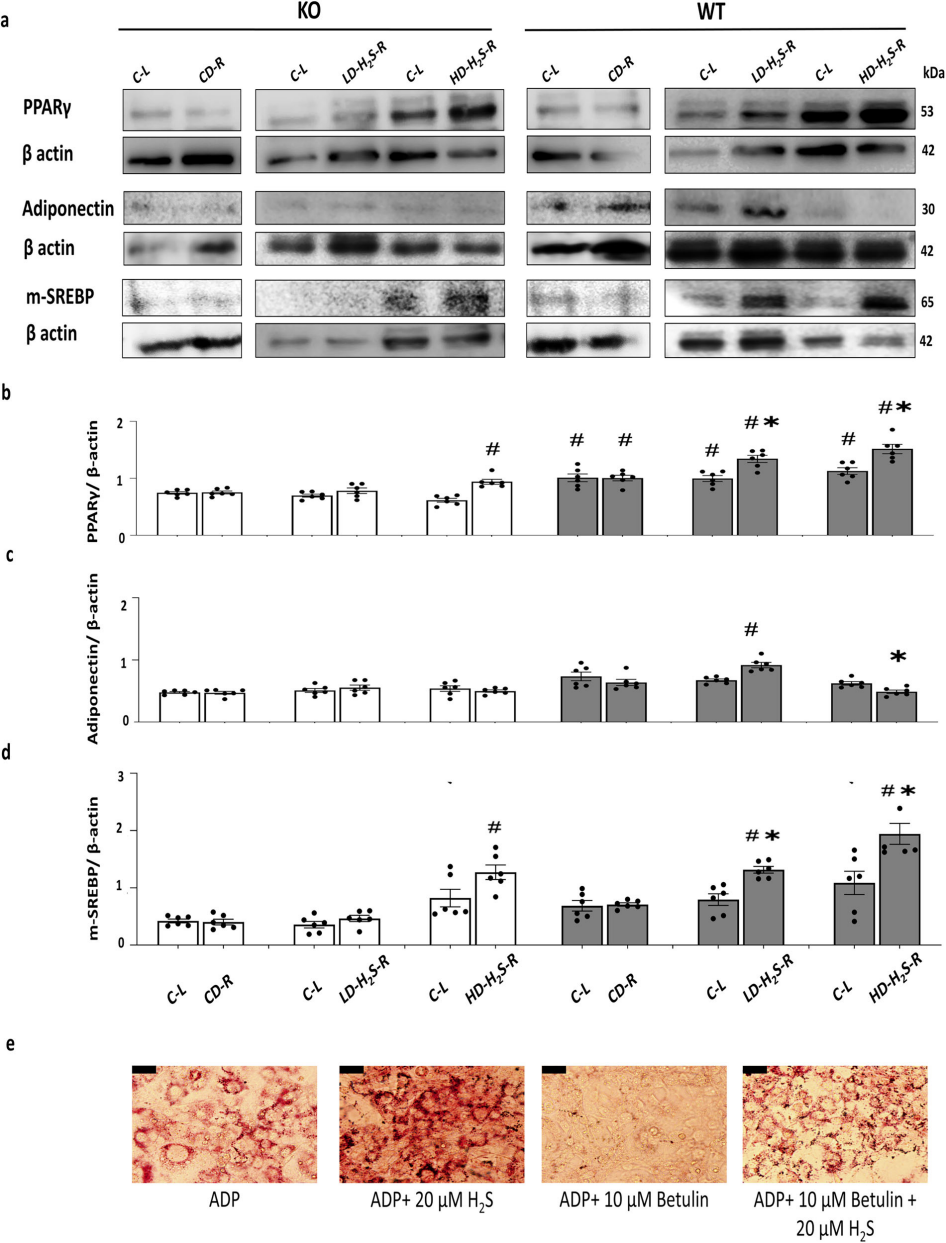
H_2_S gel regulated expression of lipogenesis and adipogenesis proteins in mouse adipose tissue. **a** Western blotting of PPARγ, m-SREBP, and adiponectin proteins in adipose tissues from WT and CSE-KO mice. **b** Relative expression levels of PPARγ protein. **c** Relative expression levels of m-SREBP protein. **d** Relative expression levels of adiponectin protein. Open columns and filled columns in (**b**–**d**) represent the tissues from WT and CSE-KO mice, respectively, after 30 days of CD injections. *p < 0.05 vs. WT CD-R and C-L; #*p* < 0.05 vs. CSE-KO CD-R and C-L. *n* = 6 for each group. m-SREBP stands for mature SREBP. **e** Oil Red O staining of mouse primary preadipocytes (*n* = 5). The red staining highlights intracellular lipid accumulation, indicative of adipogenic differentiation. ADP: adipogenic media; Betulin: SREBP inhibitor. Scale bar - 50 µm scale. The error bars in this figure represent standard error.

HD-H_2_S injections upregulated the expression of PPARγ in CSE-KO mouse adipose tissues (Fig. 5a, b). HD-H_2_S injection upregulated the expression of mature SREBP (mSREBP) in CSE-KO adipose tissues (Fig. 5a, c). HD-H_2_S and LD-H_2_S upregulated PPARγ and mSREBP expression in WT adipose tissues (Fig. 5a, c). An increased expression of adiponectin by LD-H_2_S, but not by HD-H_2_S, was noted in WT adipose tissues (Fig. 5a, d). In contrast, HD-H_2_S significantly decreased adiponectin expression in WT adipose tissues (Fig. 5a, d). PPARγ expressions was higher in WT Ctrl adipose tissue than in CSE KO Ctrl adipose tissue. CD injection did not cause any change in the expression levels of adipogenic or lipogenic marker proteins in both WT and KO adipose tissues (Fig. 5a–d).

### H_2_S promotes adipogenesis through an SREBP-dependent pathway in vitro

To determine whether H_2_S-induced lipid accumulation in adipocytes depends on SREBP activation, primary mouse preadipocytes were treated with adipogenic media (ADP), 20 µM H_2_S, 10 µM Betulin (a pharmacological SREBP inhibitor), or a combination of H_2_S and Betulin, and assessed by Oil Red O staining (Fig. 5e). Cells treated with ADP alone exhibited moderate lipid accumulation, as indicated by intracellular red staining. H_2_S treatment significantly enhanced lipid accumulation compared to the ADP group, confirming its pro-adipogenic effect. In contrast, Betulin-treated cells showed markedly reduced lipid staining, consistent with SREBP inhibition suppressing adipogenic differentiation. Notably, co-treatment with H_2_S and Betulin partially restored lipid accumulation compared to Betulin alone, suggesting that exogenous H_2_S can counteract the inhibitory effects of SREBP suppression. These findings support the hypothesis that H_2_S promotes adipogenesis at least partially through SREBP-dependent mechanisms.

### The effects of H_2_S gel on lipid accumulation in vivo

**Fig. 6:**
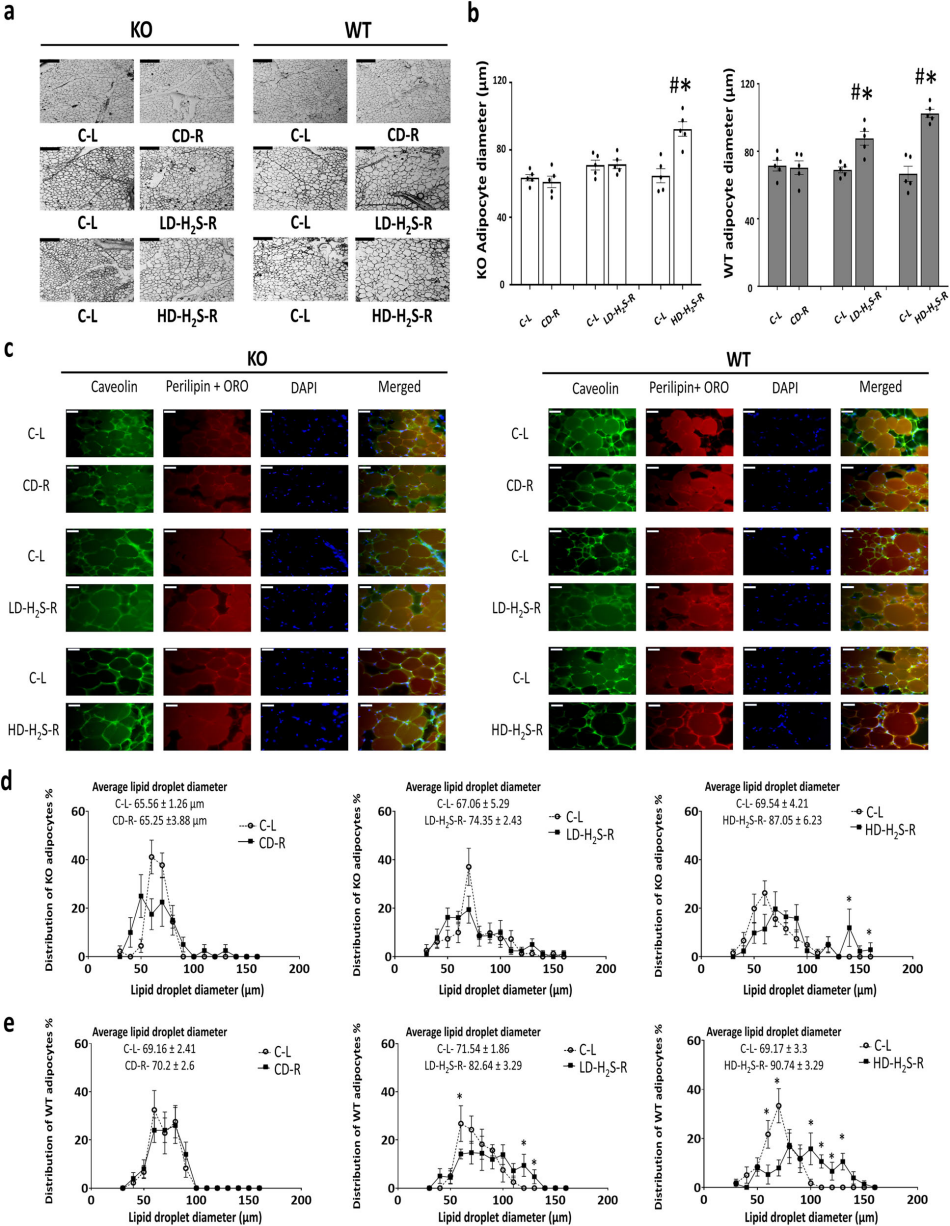
H_2_S gel increased the size of adipocytes and lipid droplets in CSE-KO and WT adipose tissue. **a** Cryosection images of adipose tissue from CSE-KO and WT mice, showing adipocyte morphology. Scale bars, 200 µm. **b** The cell diameters of adipocytes from CSE-KO and WT mice treated with CD-R, LD-H_2_S-R or HD-H_2_S-R, or no treatment (C-L). *p < 0.05 vs. WT CD-R and C-L. #*p* < 0.05 vs. CSE-KO CD-R and C-L. **c** Representative images of adipose tissue stained with Oil Red O to visualize lipid accumulation (red, lipid droplet marker), along with immunofluorescence staining for Perilipin (green, lipid droplet coating protein), and DAPI (blue, nuclear stain). Merged images were used to assess lipid droplet morphology and size. Scale bars, 50 µm. Frequency distribution of lipid droplet size from CSE-KO (**d**) and WT (**e**) mice using ImageJ software. *n* = 6 independent replicates, each replicate is the average of 10 images. *p < 0.05 vs. respective C-L. The error bars in this figure represent standard error.

No significant change in adipocyte diameter was observed in adipocytes from CSE-KO mice that received LD-H_2_S injections (Fig. 6a, b). In HD-H_2_S injected WT adipose tissue, and CSE-KO tissues, hypertrophic adipocytes were observed with the cell diameter of 89.61 ± 3 µm and 81.56 ± 5 µm, significantly larger than those from non-injected (Ctrl) and the CD-injected WT adipose tissues (Fig. 6a, b).

The frequency distribution of lipid droplet diameters in KO and WT adipose tissues reveals distinct trends following H_2_S gel injection. HD-H_2_S significantly increased lipid droplet sizes, particularly in WT adipocytes. About 41% of lipid droplets in HD-H_2_S injected WT mouse adipocytes fell in the diameter range of 100–150 µm, whereas non-treated WT cells only had about 1.7% of lipid droplets in the same diameter range (Fig. 6c, e). The most lipid droplets in non-treated CSE KO adipocytes had the diameter in the range of 60–80 µm (Fig. 6c, d) whereas the HD-H_2_S-treated KO cells exhibited about 22.1% of lipid droplets in the diameter range of 100–150 µm. However, the increase in lipid droplet size was less pronounced in CSE KO mice treated with LD-H_2_S, which aligns with lower lipid accumulation observed in these mice (Fig. 4b). These observations reveal that H_2_S increase adipocyte size as well as lipid droplet size with a more pronounced impact on WT mice.

### The effects of H_2_S gel on perilipin expression and HSL activation in vivo

**Fig. 7:**
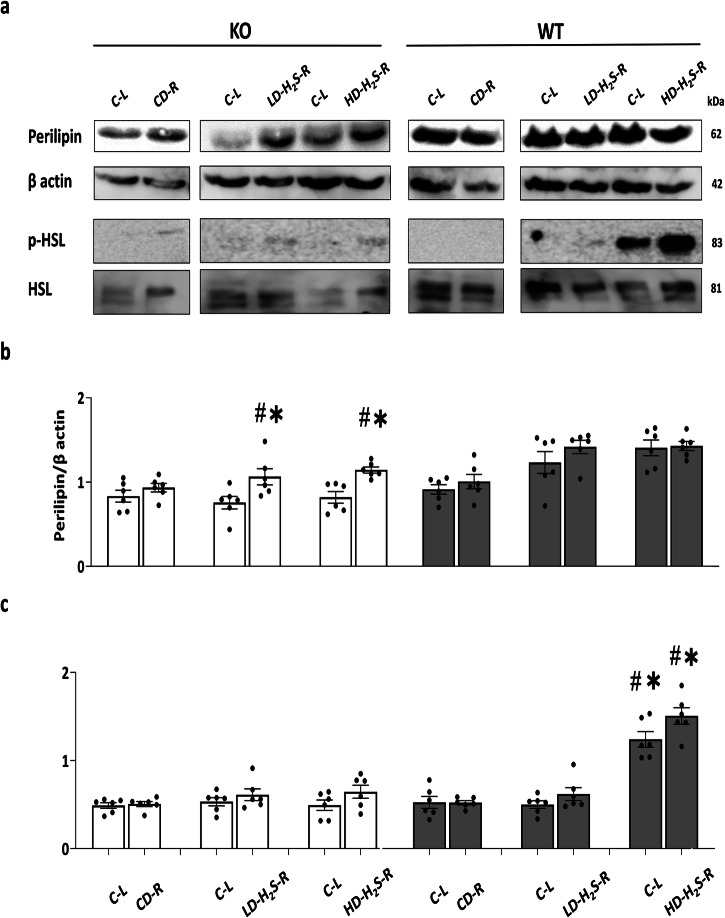
The effects of H_2_S gel on perilipin expression and HSL phosphorylation. **a** The expression of perilipin, HSL, and pHSL proteins in adipose tissue from WT and CSE-KO mice after CD-R, LD-H_2_S or HD-H_2_S injections and their controls (C-L). **b** The expression levels of perilipin. **c** The expression levels of pHSL/HSL in subcutaneous adipose tissue from CSE-KO and WT mice after 30 days of LD-H_2_S or CD injections. *p < 0.05 vs. respective C-L and CD-R; #*p* < 0.05 vs. CSE-KO CD-R and C-L, *n* = 6 for each group. HSL stands for hormone-sensitive lipase; pHSL stands for phosphorylated HSL. The error bars in this figure represent standard error.

HD-H_2_S and LD-H_2_S injection significantly increased perilipin expression in CSE-KO adipose tissues (Fig. 7a, b). While Perilipin expression remained the same in WT adipose tissues following injections of LD-H_2_S and HD-H_2_S (Fig. 7a, b). In contrast, LD-H_2_S and HD-H_2_S had no effect on the phosphorylation of HSL (pHSL) in CSE-KO adipose tissues (Fig. 7a, c). HD-H_2_S injection significantly increased pHSL level in WT adipose tissues (Fig. 7a, c). There were no discernible changes in perilipin or pHSL expressions in CSE-KO and WT adipose tissues that received CD injection or in the tissues from non-injected sides of CSE-KO and WT mice.

### The effects of H_2_S gel on macrophage infiltration

**Fig. 8:**
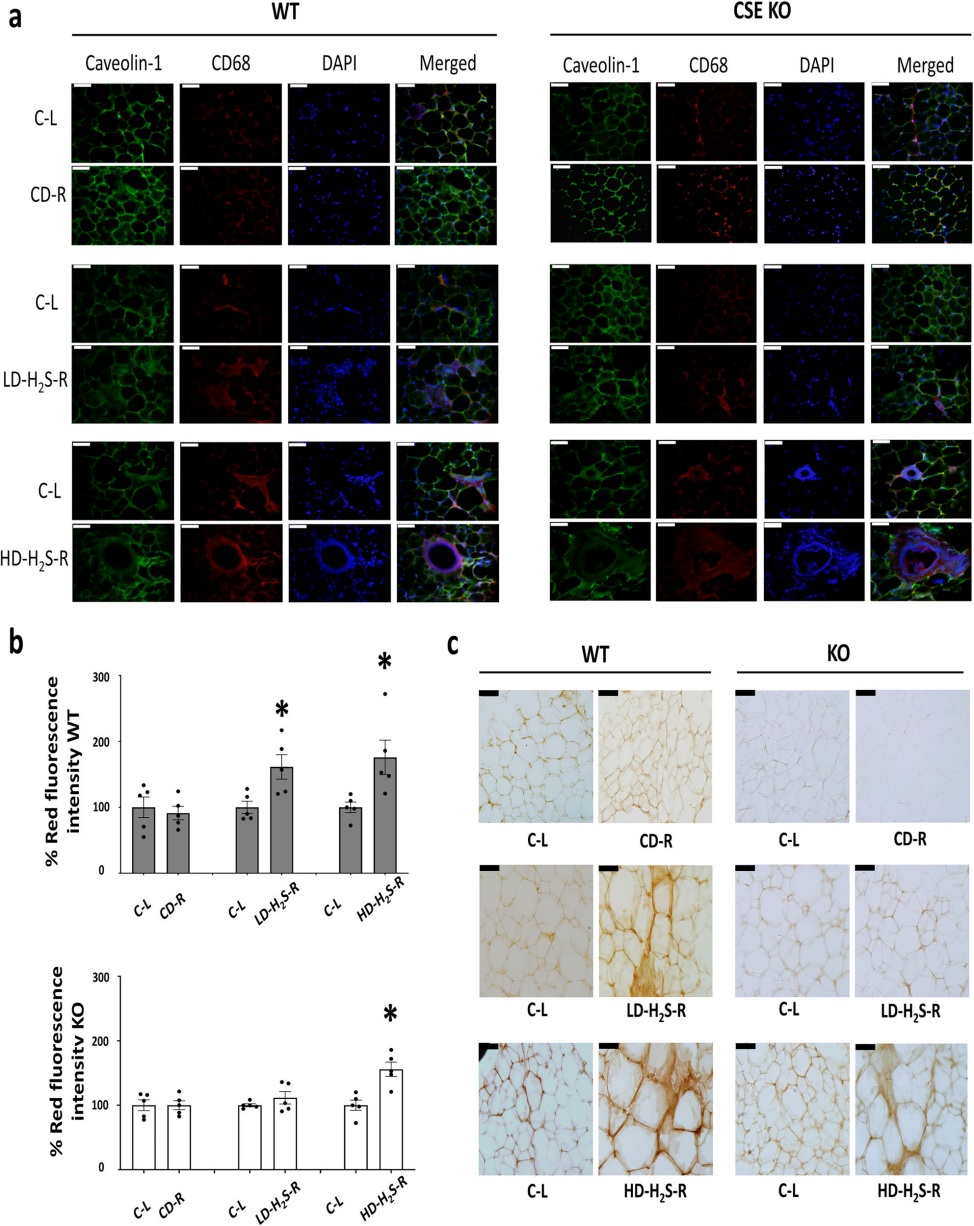
Immunofluorescence staining and quantification of macrophage infiltration in adipose tissue from WT and CSE KO mice. **a** Representative immunofluorescence images of adipose tissue sections from WT and CSE KO mice stained for Caveolin-1 (green, marking lipid raft domains), CD68 (red, a macrophage marker), and DAPI (blue, staining nuclei). Scale bars: 50 μm. **b** Quantification of red fluorescence intensity for CD68 in WT and CSE KO adipose tissue (*n* = 5). *p < 0.05 vs. respective C-L and CD-R. **c** Immunohistochemistry images of adipocytes marked by CD68 macrophages (dark brown). Scale bars: 50 μm. The error bars in this figure represent standard error.

CD68 immunofluorescence staining was used to assess macrophage infiltration in adipose tissue from wild-type (WT) and CSE-KO mice (Fig. 8). In WT mice, LD-H_2_S and HD-H_2_S treatment increased CD68 fluorescence intensity compared to the control and C-E4-treated groups (Fig. 8a, b). Quantification showed a significant increase in corrected total cell fluorescence in LD-H_2_S and HD-H_2_S -treated groups (*p* < 0.05). In CSE-KO mice, LD-H_2_S did not significantly alter CD68 fluorescence, whereas HD-H_2_S treatment led to a significant increase (Fig. 8c, d). Control and C-E4 groups showed no significant differences in fluorescence intensity.

### The effects of H_2_S gel on collagen accumulation and fibrosis

**Fig. 9:**
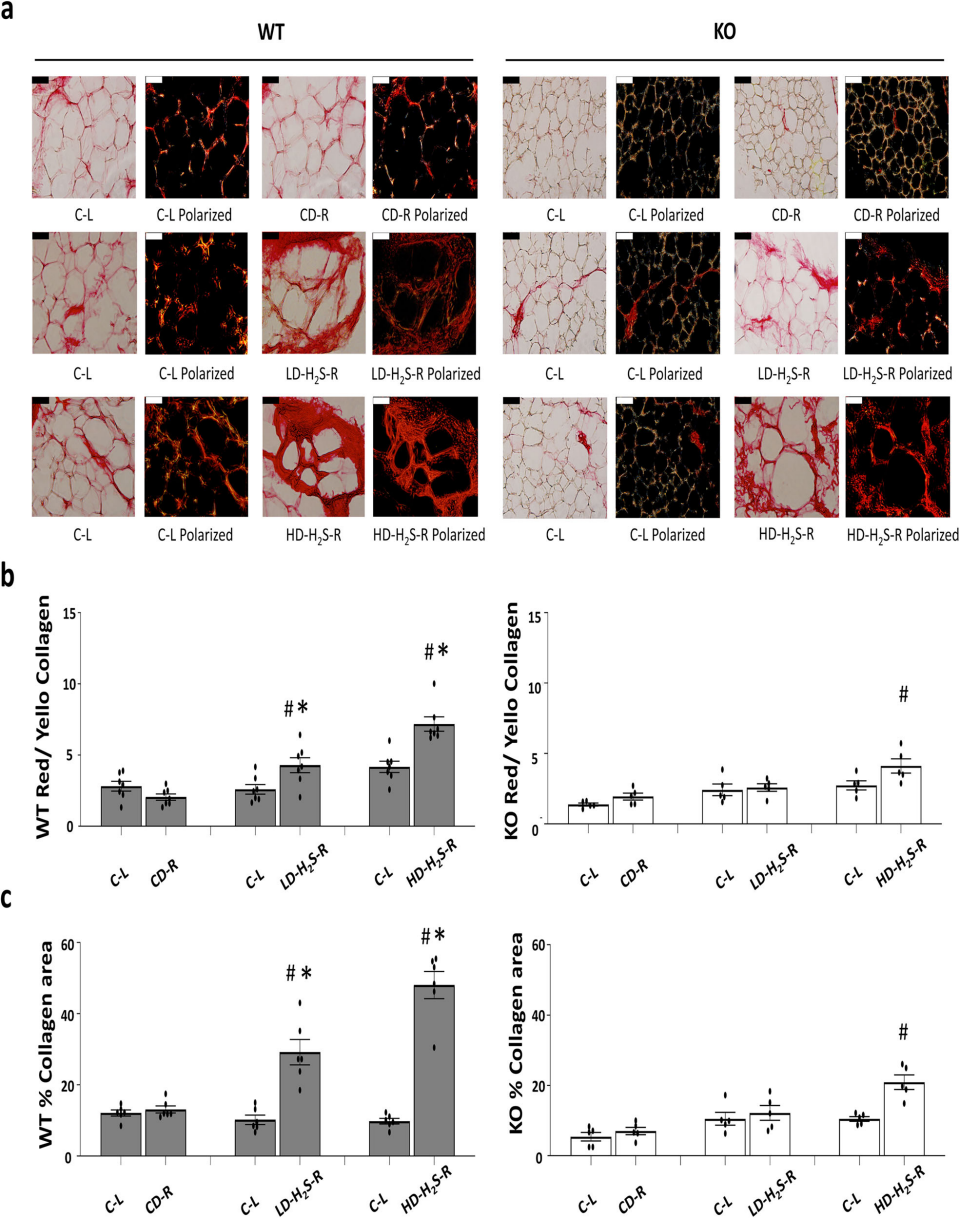
Picrosirius red staining of WT and CSE KO Adipose tissue to assess collagen deposition and fibrosis. **a** Representative images of adipose tissue stained with Picrosirius Red. The left images show regular light microscopy, while the right images show polarized light images, where collagen fibers appear as red or yellow. Red collagen represents thicker, older collagen fibers, while yellow collagen indicates thinner, newly formed collagen fibers. Scale bars = 50 μm. **b** The ratio of red/yellow collagen fibers in WT and KO mouse adipose tissue sections, analysed based on polarized image. **c** The total collagen area (%) in the adipose tissue of WT and KO mice. *p < 0.05 vs. WT C-L and CD-R; #*p* < 0.05 vs. CSE-KO C-L and CD-R, *n* = 5 for each group. The error bars in this figure represent standard error.

Picrosirius Red staining was used to evaluate collagen deposition in adipose tissue. Treatment with exogenous H_2_S induced fibrosis in a dose-dependent manner. In WT mice, both HD-H_2_S gel and LD-H_2_S gel increased collagen accumulation, with a more pronounced effect observed at higher H_2_S concentrations (Fig. 9a, b). In CSE-KO mice, LD-H_2_S did not induce significant fibrosis, whereas HD-H_2_S resulted in a mild increase in collagen deposition (Fig. 9a, c). Collagen deposition was higher on the Right side compared to the Left, suggesting localized effects of treatment on ECM remodeling. WT and CSE-KO control groups showed comparable ECM composition, with no significant differences in collagen deposition (Fig. 9a, b).

## Discussion

A plethora of physiological and pathophysiological effects of H_2_S has been reported. Increased H_2_S concentration is beneficial for the health of numerous systems, such as the cardiovascular, respiratory, neuronal, or reproductive systems^7–9,13^. On the other hand, over-production of H_2_S may constitute a pathogenic factor for diabetes^18–21^, obesity^10,12^, and certain types of inflammation or cancers^22^. These differential effects of H_2_S on specific targets render the interpretation of the outcome of systematic H_2_S administration perplex and inconclusive. Administration of H_2_S salts, such as NaHS and Na_2_S, would deliver H_2_S that would distribute to and disappear from the whole body. The frequently employed H_2_S-releasing compound, GYY4137, is a water-soluble salt that liberates H_2_S in a slow process^23,24^. It is administered through daily injection over many days or weeks, and the released H_2_S would disperse widely and enter the circulation. Other available means for H_2_S delivery, such as H_2_S-prodrugs or H_2_S hybrids with other known compounds, face the same challenge of reaching the specific targets without the system-wide spill-over effect^24^. Our results from the current study offer one solution for this challenge for localized delivery and retention of H_2_S while achieving regional effect.

The SATO-Ile-Ala-Val-Glu-Glu (SATO-IAVE_2_) gelator is a peptide sequence linked to an *S*-aroylthiooxime (SATO), forming an aromatic peptide amphiphile (APA) hydrogel^17,25–27^. The gelation of H_2_S gel was initiated by calcium ions that crosslink the peptide units through salt bridges, thus aiding the formation of hydrogel^17,25–27^. H_2_S gel is a biodegradable gel, and as cysteine reacts with the SATO unit, H_2_S is released, and the gel degrades (Fig. 1)^17,25–27^. The 3D network forms a depot in the form of a hydrogel near the site of action, ensuring that the drug is delivered precisely where it is needed. This technique limits the delivery of H_2_S to a specific localized area, which is the adipose tissue in our case. Moreover, the localized H_2_S gel can provide continuous and efficient drug delivery. A control peptide hydrogel of a similar chemical structure but with an oxygen atom in place of the sulfur was also studied (CD) as a material control that was incapable of H_2_S release.

H_2_S gel was confirmed to be safe in our current study as it showed no adverse effects on mice behavior or mortality rate, neither causing any change in the body weights of WT and CSE-KO mice. H_2_S gel was absent in or near adipose tissue after 30 days of injections, indicating that the gel had degraded before the subsequent injection. This aligns with our in vitro H_2_S gel release studies, which predicted that the gel would degrade before the next injection given to the mice in vivo (6 days). In vitro studies revealed that H_2_S gel provided a controlled slow release of H_2_S for up to 72 h upon contact with cysteine, whether endogenously or exogenously supplied. The H_2_S concentration was found to be highest in adipose tissue after 12 h of injection during in vitro testing. Similarly, in a 30-day in vivo study on mice, adipose tissue exhibited a high H_2_S concentration, while no significant increase was observed in H_2_S concentration in the plasma. These results indicate that H_2_S release was restricted to adipose tissue, effectively targeting the delivery and retention of H_2_S to this specific site. Elevated exogenous H_2_S levels in regional adipose tissues in mice led to the accumulation of lipids in adipocytes, validating the effectiveness of H_2_S gel in achieving localized H_2_S delivery and localized functional changes.

Adipose tissue plays a critical role in regulating lipid metabolism and energy storage, but excess body fat, often associated with obesity, can disrupt this balance, leading to metabolic disorders, inflammation, insulin resistance, and conditions like type 2 diabetes and cardiovascular disease^1–5^. The three key stages of lipid metabolism in adipose tissue are adipogenesis, lipogenesis, and lipolysis. Adipogenesis involves the differentiation of preadipocytes into mature adipocytes, primarily driven by insulin and transcription factors. These mature adipocytes esterify fatty acids and glycerol to form triacylglycerol (TAG). Lipogenesis encompasses the synthesis of fatty acids and their incorporation into various lipids. De novo lipogenesis, a subset of this process, occurs primarily in the liver during energy surplus, synthesizing fatty acids from non-lipid precursors like glucose^6–8^. Lastly, lipolysis involves the enzymatic breakdown of stored TAGs in adipocytes, releasing free fatty acids and glycerol into the bloodstream, with HSL playing a crucial role in initiating triglyceride hydrolysis^8,28^.

Our previous in vitro experiments proved that H_2_S increased the rate of adipogenesis and elevated lipid levels in cultured adipocytes^12,14^. To investigate the in vivo adipogenic and lipogenic effects of H_2_S, we injected H_2_S gel hydrogel into mouse subcutaneous adipose tissue (SAT). While other fat depots, such as visceral adipose tissues, have distinct endocrine functions and hormone secretion, SAT stands out quantitatively as the largest adipose depot with a significant contribution to lipid storage and release. This makes SAT an ideal target for studying the impact of H_2_S on adipose tissue dynamics^29^.

In WT mice, we observed an increase in adipose tissue lipid content after having injected low- and high- doses of H_2_S gel. In CSE-KO mice, only the high dose of H_2_S gel increased lipid content of adipose tissues. The major difference between WT and CSE-KO mice is the minimal level of endogenous H_2_S in the latter group, which may thus elevate the threshold level of exogenous H_2_S to increase local lipid levels. Previous in vitro cellular studies have indicated a specific concentration range where exogenous H_2_S exhibited the adipogenic effect^10,30^. Moreover, exogenous H_2_S may stimulate additional endogenous H_2_S release by increasing CSE expression as reported in the plasma, ischemic heart tissue^31^, and mammalian myocardial cells^32^. This exogenous H_2_S-induced reinforcement of endogenous H_2_S production was shown in the current study where H_2_S gel increased CSE expression and H_2_S production in WT adipose tissues, but not in CSE-KO adipose tissues due to their lack of CSE expression (Fig. 4).

We then asked whether H_2_S gel can upregulate the expression of other H_2_S-generating enzymes in addition to CSE. H_2_S can be produced by at least three enzymes, i.e., CSE, cystathionine beta synthase (CBS), and 3-mercaptopyruvate sulfurtransferase (MST) in vivo^9–11^. In mouse adipose tissue, CSE was the most abundantly expressed, followed by MST and CBS^14^. Under laboratory conditions, the cystathionine aminotransferase-MST pathways produce H_2_S with optimal alkaline conditions and high cysteine concentration^10^. However, under more physiological conditions, MST fails to produce meaningful H_2_S in rat liver and kidney^10,33^. Moreover, the unstable nature of 3-mercaptopyruvate poses a significant challenge as it is the only sulfur donor for MST, and its presence in the body is not yet directly detected^10^. Although MST and CBS expressions were observed in CSE-KO mouse adipose tissues (Fig. 4), H_2_S gel injections did not change their expression nor their H_2_S production rates (Figs. 2a and 4a).

Consistent with our previous in vitro studies^12^, the current study demonstrated that increased local H_2_S concentration stimulated adipogenesis and lipogenesis, leading to larger lipid droplets and increased lipid content in mouse adipose tissue. High doses of H_2_S gel led to higher local H_2_S concentration and increased hypertrophic adipocytes with larger lipid droplets in the injected mouse adipose tissues.

The current study revealed that low doses of H_2_S gel had no effect on lipid accumulation in CSE-KO adipose tissue in vivo. However, our previous in vitro experiments showed increased adipogenesis at NaHS concentrations ranging from 10–100 µM in CSE-KO adipocytes^12^. This discrepancy may stem from the slowly delivered H_2_S not reaching the necessary concentration threshold to exert its physiological effects in vivo. Furthermore, our current in vivo study shows that WT adipocytes injected with HD-H_2_S exhibited hypertrophy and reduced pHSL/HSL expression, but this effect was not observed in H_2_S-induced hypertrophic adipocytes in vitro^12^. This difference may be related to the diverse effects of H_2_S on matured adipocytes, preadipocytes, and other types of cells in vivo.

The mechanisms for H_2_S-induced local lipid accumulation revealed in the current in vivo study aligns well with our previous in vitro research^12^. The expression levels of PPARγ, a key adipogenic marker and master regulator of adipogenesis and lipid storage^34,35^, are upregulated in both WT and CSE-KO mice by H_2_S gel injections. SREBP belongs to the family of lipogenic transcription factors governing the expression of various lipogenic enzymes. Full SREBPs (fSREBPs) are synthesized and bound to the rough endoplasmic reticulum membrane as full-length precursor proteins. Upon activation, fSREBPs undergo sequential cleavage yielding matured SREBP (mSREBP), which enters the nucleus to activate transcription by binding to specific sterol response elements in the promoter regions of lipogenic genes^35–37^. The activation of SREBP to mSREBP serves as an indicator of the tissue’s lipogenic activity. Using mSREBP inhibitors in mouse primary preadipocytes, we found that H_2_S treatment significantly increased lipid accumulation (via Oil Red O staining), which was abolished by pre-treatment with the inhibitor. This suggests H_2_S promotes lipid biosynthesis through mSREBP reactivation. Although our focus remains on the pharmacological effects of localized H_2_S delivery via H_2_S gel, these findings propose a potential mSREBP-dependent mechanism underlying H_2_S-induced lipid accumulation. The similar upregulation of mSREBP by H_2_S has been shown in the previous studies on cultured 3T3L1 cells in vitro^32^.

Adiponectin, a hormone with significant roles in promoting insulin sensitivity, energy balance, and inflammation, is upregulated by PPARγ through direct binding to conserved cis-acting regulatory DNA elements in the promoter region of the adiponectin gene^34,35,38,39^. In conditions like obesity or when adipocytes undergo hypertrophy, the normal upregulation of adiponectin by PPARγ is hindered, leading to reduced adiponectin expression. This disruption primarily stems from chronic low-grade inflammation within adipose tissue in obesity, marked by the release of inflammatory cytokines such as TNF-α and IL-6^39^. These cytokines can disrupt PPARγ‘s function and its capacity to promote adiponectin expression by competing with PPARγ for coactivators that are essential for its transcriptional activity. The injections of low-dose H_2_S gel upregulated adiponectin expression in WT adipose tissues. The stimulatory effect of H_2_S on adiponectin expression was previously shown with the cultured primary adipocytes^12^ as well as in another study on 3T3L1 cells^40^. In contrast, HD-H_2_S injected WT adipose tissue with both larger lipid droplets and larger average adipocyte diameter exhibited downregulated adiponectin expression, indicating putative toxic effects of high doses of exogenous H_2_S in the presence of endogenous H_2_S production^39,41^.

Perilipin coats lipid droplets, preventing their breakdown under normal conditions. When energy deficits occur, protein kinase A (PKA) phosphorylates perilipin, dissociating it from the lipid droplets. This action enables HSL and adipose triglyceride lipase (ATGL) to breakdown the droplets into fatty acids and glycerol^28,42–47^. H_2_S-induced lipid accumulation involves the formation of large lipid droplets and inhibit perilipin1-mediated lipolysis^42–47^. The presence or increase in perilipin indicates the maturation, enlargement, and fusion of adipocyte lipid droplets. In our previous cell study in vitro^12^, H_2_S treatment during adipocyte differentiation did not affect free fatty acid concentration or HSL phosphorylation, suggesting that H_2_S-stimulated adipogenesis and lipogenesis are independent of lipolysis. However, the current in vivo mouse study showed that a high dose of H_2_S gel significantly increased HSL phosphorylation in WT mouse adipose tissues, which could be due to the significant increase in lipid accumulation in WT adipose tissue. HSL is an indicator of lipolysis since it is the primary enzyme responsible for mobilizing free fatty acids from adipose tissue^28,46^. In CSE-KO mouse adipose tissue, perilipin levels increased with H_2_S gel injections but not in WT adipose tissue (Fig. 7). One reason for these divergent results between in vitro and in vivo studies may be the complex regulation of lipolysis, influenced by factors such as the nervous system, hormones, and paracrine/autocrine factors, which may not be completely recapitulated in vitro. Furthermore, the enlargement of adipocytes causes higher storage of triglycerides. Yet, the expression level of perilipin does not necessarily increase, because perilipin predominantly controls lipolysis, not triglyceride/lipid synthesis^44^.

Our results demonstrate that H_2_S influences macrophage infiltration and fibrosis in a concentration-dependent manner in mouse adipose tissues. WT tissues exhibited greater macrophage presence compared to CSE-KO tissues, suggesting that endogenous CSE-derived H_2_S promotes immune cell recruitment. Picrosirius red staining revealed that increased H_2_S levels were associated with enhanced collagen deposition and fibrosis, but only at higher concentrations. Specifically, in WT mice, both LD-H_2_S and HD-H_2_S increased collagen accumulation, with a more pronounced effect at higher H_2_S concentrations. This increase was accompanied by a higher red/yellow collagen ratio in WT adipose tissues, particularly in the HD-H_2_S group, indicating enhanced collagen crosslinking and maturation. Red collagen represents newly synthesized, loosely packed fibers, while yellow collagen corresponds to mature, highly crosslinked fibers that contribute to fibrosis. In contrast, CSE-KO mice treated with LD-H_2_S showed no significant changes in fibrosis, while HD-H_2_S induced a mild increase, suggesting that high-dose exogenous H_2_S may trigger ECM remodeling. However, the lower red/yellow collagen ratio in KO mice reinforces the idea that endogenous H_2_S is necessary for robust collagen crosslinking and ECM remodeling.

Immunohistochemical staining for CD68 showed that H_2_S gel treatment increased macrophage infiltration in WT mice, with a stronger effect at higher doses, suggesting a dose-dependent inflammatory response to exogenous H_2_S. In contrast, CSE-KO control mice exhibited lower macrophage presence, indicating that endogenous H_2_S plays a role in basal macrophage regulation. While LD-H_2_S did not significantly alter macrophage infiltration in CSE-KO mice, HD-H_2_S led to a moderate increase, suggesting that exogenous H_2_S at high doses can partially restore macrophage recruitment in the absence of CSE.

Previous studies suggest that H_2_S regulates ECM remodeling and immune cell recruitment in a dose-dependent manner^48,49^. The lack of significant fibrosis differences between WT and CSE-KO control groups suggests that CSE-derived H_2_S does not actively regulate ECM composition under normal physiological conditions. Instead, H_2_S primarily modulates immune cell recruitment, as reflected in the higher macrophage presence in WT control mice. The red/yellow collagen ratio findings further support this, as WT mice exhibited a clear dose-dependent increase, whereas KO mice did not show a significant response.

Our findings suggest that at physiological levels, H_2_S primarily influences immune homeostasis, while at higher concentrations, it drives immune activation and ECM remodeling. Potential mechanisms include oxidative stress modulation, macrophage polarization shifts toward a pro-inflammatory M1 phenotype, and interactions with cytokine-ECM signaling pathways^50^.

## Conclusion

This study demonstrates that localized, slow-releasing delivery of H_2_S via the injectable H_2_S hydrogel promotes lipid accumulation, adipocyte hypertrophy, and tissue remodeling in subcutaneous adipose tissue in vivo. By comparing WT and CSE-KO, we highlight the physiological role of endogenous H_2_S in adipose regulation and confirm a dose-dependent threshold effect for exogenous H_2_S. While our study emphasizes the pharmacological impact of localized H_2_S delivery, mechanistic insights—particularly involving adipogenic regulators like SREBP—were primarily supported by in vitro analyses. Overall, our findings offer a platform for targeted H_2_S modulation in metabolic tissues and suggest that precise control of H_2_S levels may be critical for maintaining adipose tissue homeostasis and preventing obesity-related dysfunction.

Impaired H_2_S metabolism is related with metabolic disorders, including obesity, insulin resistance, and inflammation^12,14,40^. At physiological levels, H_2_S supports adipogenesis and energy homeostasis, but excessive accumulation of H_2_S disrupts lipid metabolism and dysregulate adipocyte phenotype. Previous studies have shown that high-fat diet (HFD) feeding of C57BL/6 J mice increases CSE expression and H_2_S production in adipose tissue, underscoring the metabolic importance of the CSE/H_2_S system^14^.

**Fig. 10:**
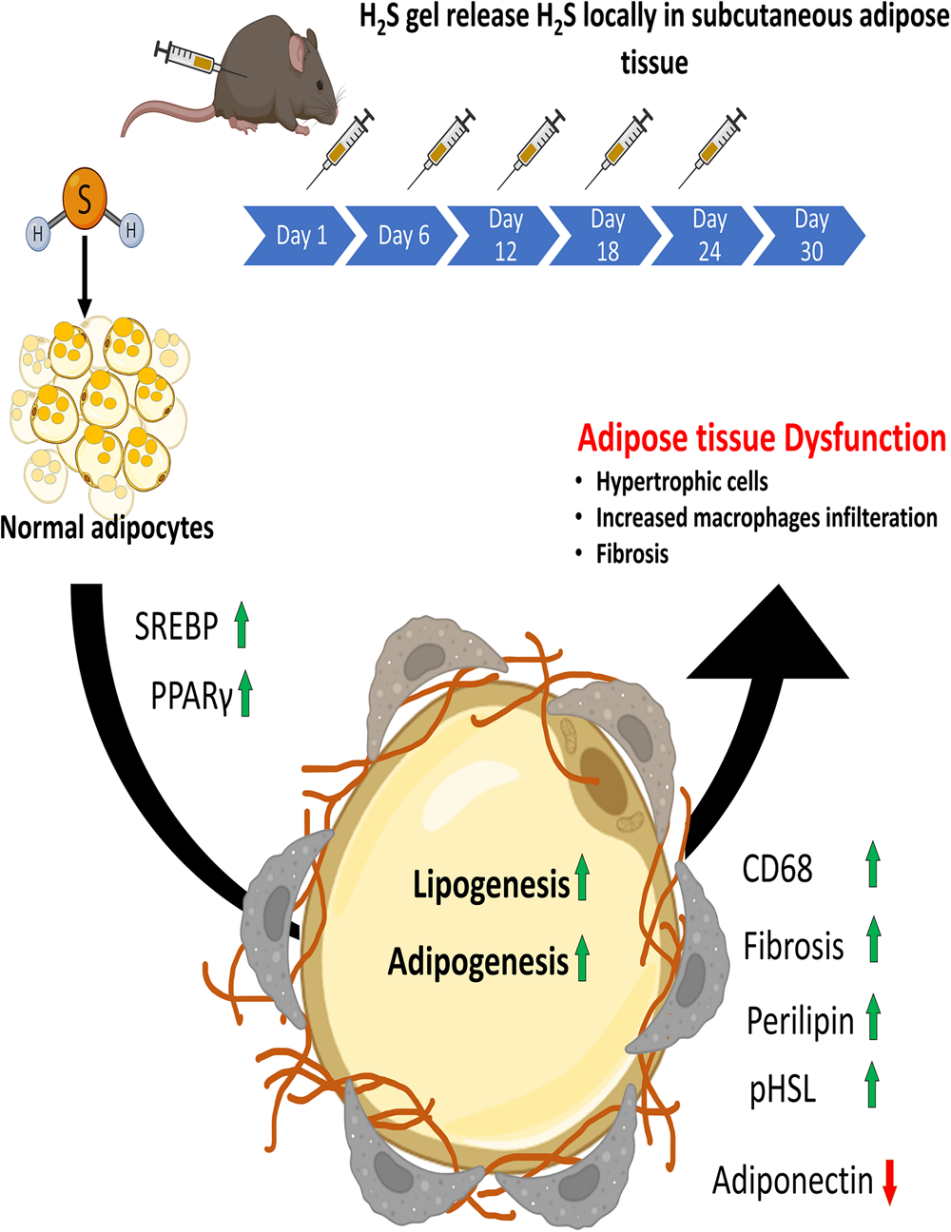
Localized delivery of H_2_S via hydrogel induces adipogenesis, lipogenesis, and adipose tissue dysfunction in vivo. Subcutaneous injections of H_2_S-releasing hydrogel release H_2_S in subcutaneous adipose tissue, which increases adipogenesis and lipogenesis by upregulation of PPARγ and SREBP. Consequently, adipocyte hypertrophy and tissue remodeling are promoted, resulting in adipose tissue dysfunction characterized by increased CD68^+^ macrophage infiltration, fibrosis, elevated perilipin and pHSL levels, and altered adiponectin expression. Created with BioRender. Verma, R. (2025) https://BioRender.com/1hrgiek.

To our knowledge, our study is the first to demonstrate that localized, slow-releasing H_2_S delivery via the injectable H_2_S hydrogel induces obesity-like remodeling of adipose tissue in vivo, including lipid accumulation, adipocyte hypertrophy, macrophage infiltration, and fibrosis (Fig. 10). We show that total H_2_S levels—both endogenous and exogenous—correlate with increased lipid content in subcutaneous adipose tissue, and that hypertrophy occurs at the highest concentrations of H_2_S. While previous cellular studies reported similar adipogenic effects of H_2_S in vitro^12,14,40^, this work provides direct in vivo evidence of site-specific H_2_S-driven adipose expansion.

Importantly, our comparative analyses using CSE-KO and WT mice reveal that endogenous H_2_S is required to sensitize adipose tissue to exogenous H_2_S, supporting a threshold effect in adipogenesis and lipogenesis^9,12,14^. CSE-KO mice showed decreased lipid accumulation, and downregulated expression of adipogenic transcription factors (PPARγ and SREBP), highlighting the multifaceted role of endogenous H_2_S in maintaining adipose tissue homeostasis^9,12,14^.

Moreover, our findings reveal that high concentrations of H_2_S induce a pathological shift, including downregulation of adiponectin and promotion of macrophage infiltration and fibrosis (Fig. 10) mirroring conditions seen in dysfunctional adipose tissue in obesity^51^. This biphasic effect of H_2_S, where low doses promote homeostasis and high doses drive dysfunction, has also been observed in cancer biology^52^. Previous studies suggest that in obese adipose tissue, despite increased CSE expression, H_2_S bioavailability is reduced due to oxidative consumption, leading to enhanced Ca^2+^ influx and M1 macrophage polarization^53^. Our results suggest that exogenous H_2_S at high doses may similarly exacerbate inflammation and immune activation in adipose tissue.

Clinically, these findings carry substantial implications. The ability to precisely modulate H_2_S levels in adipose tissue using localized, biodegradable hydrogels like H_2_S gel opens therapeutic avenues for obesity and related metabolic diseases. Tissue-specific H_2_S inhibition or scavenging strategies could help restore adipose tissue function in the presence of excess of H_2_S, while controlled delivery could support regeneration where H_2_S is deficient. Moreover, the platform’s adaptability offers potential for organ-targeted interventions in other H_2_S-linked conditions beyond adipose biology.

The study further suggests that H_2_S-regulated adipogenic pathways are conserved across species and may be relevant to human adipose biology. However, additional studies in diverse animal models and human tissues are needed to confirm the broader applicability and therapeutic potential of targeting the CSE/H_2_S axis.

Ultimately, our study provides critical insight into the dual physiological and pathological roles of H_2_S in adipose tissue, while laying a strong foundation for precision-based therapeutic strategies that harness or restrain H_2_S in a tissue-specific and clinically translatable manner.

## Material and Methods

### Preparation procedure for H_2_S gel, control gel, and their H_2_S release in the presence of adipose tissues

An aromatic peptide amphiphile (APA) hydrogel was used, featuring an *S*-aroylthiooxime (SATO) functional group at the N-terminus of the IAVE_2_ peptide sequence Ile-Ala-Val-Glu-Glu (Fig. 1). The peptide was synthesized in a similar manner to a related peptide^25^. When combined with 10% CaCl_2_, the APA forms a biodegradable hydrogel inside the tissue. The SATO unit of the APA releases H_2_S upon contact with thiols like cysteine and glutathione^26^. A control APA, called Control-IAVE_4_ (CD), has similar rheological properties to SATO-IAVE_2_ (H_2_S gel) but has an oxygen atom in place of the sulfur and cannot release H_2_S^25,26^. CD and two doses of SATO-IAVE_2_ were used, SATO-IAVE_2_-Low (LD-H_2_S; 39 µmol/kg H_2_S) and SATO-IAVE_2_-High (HD-H_2_S; 78 µmol/kg H_2_S).

### Animal and tissue preparation

Wild-type (WT) mice were maintained on C57BL/6J × 129SvEv background. In-house-bred homozygous CSE-KO mice were generated after backcrossing of at least 10 generations of heterozygous of CSE-KO mice with WT mice with the same genetic background^54^. All WT and CSE-KO mice were verified by genotyping and used in this study. Each group of WT and their matching CSE-KO mice were from the same generations. WT and CSE-KO mice were maintained on standard rodent chow and had free access to food and water. Mice were monitored daily for general health, including activity level, grooming behavior, body weight, and signs of distress. No adverse effects or signs of pain or illness were observed throughout the study duration. All animal experiments conducted were in compliance with the Guide for the Care and Use of Laboratory Animals, published by the US National Institutes of Health (NIH Publication No. 85-23, revised 1996), and were approved by the Animal Care Committees of York University and Laurentian University in Canada.

Only male mice aged 5 weeks were included to reduce variability due to sex and developmental stage. No additional inclusion or exclusion criteria were applied. Male mice of 5 weeks old received injections at intervals of every 6 days over a 30-day period. To minimize pain, stress, and discomfort, mice were lightly anesthetized using isoflurane during each injection procedure, in accordance with approved animal care protocols. We have complied with all relevant ethical regulations for animal use.

H_2_S gel was prepared as previously described^17^ by mixing 63 µL of H_2_S gel solution (0.01 mg/mL in saline) and 7 µL of 10 wt.% CaCl_2_ solution in saline. H_2_S gel CaCl_2_ solution was quickly injected into the subcutaneous adipose tissue (SAT) located on the right rear flank side of the mouse body, between the ribcage and the hind legs. The SAT on the left rear flank side served as a non-injected control. At the end of the experiment, the mice were euthanized. Regional SAT from both the left and right rear flank at the injection sites were removed, weighed, and immediately frozen in liquid nitrogen and stored at −80 °C.

### Measurement of H_2_S levels under different conditions

#### In vitro H_2_S release from aromatic peptide amphiphile

A Reaction Mixture was prepared by mixing 63 µL of H_2_S gel solution and 7 µL of CaCl_2_ solution, prepared as described above, with 2 mL of 50 mM sodium carbonate buffer (pH 8.8) containing 400 mg adipose tissues and protease inhibitors and 10% penicillin-streptomycin solution (Sigma-Aldrich). A control for this reaction mixture had identical mixture components except for the absence of H_2_S gel solution.

At predetermined intervals (0, 6, 12, 24, 48, and 72 h), 200 µL of adipose tissue lysate were withdrawn from the Reaction Mixture, named as Measure Unit here. To each Measure Unit, 250 µL of 1% zinc acetate and 50 µL of double-distilled water were added and mixed. Then, 50 µL of *N*, *N*-dimethyl-*p*-phenylenediamine sulphate (DPD) and 50 µL of FeCl_3_ were added to the fully mixed Measure Unit in the dark for 20 min. The released sulfide reacted with DPD in the presence of FeCl_3_ under acidic conditions, yielding methylene blue. Subsequently, 50 µL of 10% trichloroacetic acid (TCA) was added to denature and precipitate all the protein in the Measure Unit, which was then centrifuged at 14000 RPM for 5 min. The supernatant was used for measuring the formation of methylene blue at an absorbance of 670 nm.

#### H_2_S levels in mouse adipose tissues

Adipose tissues, the liver, and spleen of 100 mg each were removed from WT mice 30 days after H_2_S gel injection and homogenized in 0.5 mL of 50 mM sodium carbonate buffer (pH 10). Blood samples were also obtained from the same mice. We mixed 200 µL of tissue lysates or 200 µL of plasma with 250 µL of 1% zinc acetate and 50 µL of double-distilled water. DPD of 50 µL and FeCl_3_ of 50 µL were then added in the dark for 20 min. Trichloroacetic acid (TCA) of 50 µL at 10% was added to denature and precipitate proteins, followed by centrifugation at 14000 RPM for 5 min. The supernatant containing methylene blue was filtered through a 20 µm filter for H_2_S measurement.

#### HPLC measurement of H_2_S level

A Dionex Ultimate 3000 U-HPLC with a diode array detector set to 660 nm (Thermo Scientific) allows a lower detection limit of 650 nM of methylene blue. Aliquots (200 µL) of the filtered sample solutions were injected into a C18 column (250 × 4.6, 5 μm, Thermo Scientific Hypersil GOLD). A 20 min gradient elution, using acetonitrile and 0.1% (v/v) trifluoroacetic acid in water as a mobile phase, was run at a flow rate of 1 mL/min. The gradient elution protocol used acetonitrile and water in various proportions, ranging from 10% to 50% acetonitrile and 50% to 90% water with 0.1% trifluoroacetic acid. Standard solutions of NaHS were prepared from stock solution with appropriate dilution in 1% zinc acetate solution. DPD and FeCl_3_ were then added and kept in the dark for 20 min to produce methylene blue. TCA was added to keep the conditions consistent with tissue samples. The methylene blue peak was detected on the HPLC chromatogram at a retention time of 12.33 min. The area under each peak was plotted against corresponding NaHS standards to construct the H_2_S calibration curve.

### H_2_S gel in vitro degradation study

Adipose tissue samples were extracted from WT mice and incubated in Dulbecco’s Modified Eagle’s Medium (DMEM, 4.5 g/L glucose, SA) supplemented with 10% fetal bovine serum (FBS) and 1% pen/strep at 37 °C in vitro. We injected H_2_S gel, prepared as described above, into the tissue in vitro, and photographs were taken to document the changes in the physical appearance of the injected H_2_S gel.

### Changes in total H_2_S levels of mouse adipose tissues

Adipose tissues were sonicated in an ice-cold PBS buffer. In a 96-well plate, 60 μL of cell lysates with an equal amount of protein (200 μg) were mixed with 10 μl of cysteine (10 mM), 10 μl of pyridoxial 5′-phosphate (2 mM), and 20 μl of double-distilled water. A lead acetate paper prepared by soaking filter paper in a 1% lead acetate solution followed by drying was placed over the well for 2 h at 37 °C in the dark. The intensity of the darkening on the lead acetate paper, reflecting H_2_S level^55,56^, was analyzed using Image J software. The percent increase/decrease in H_2_S generated from our H_2_S gel injected tissues was relative to the WT control (WT Ctrl) multiplied by 100.

### Lipid extraction from adipose tissue

The SAT fat pads from both the right (injection site) and left (control site) rear flank regions were removed, and the Bligh and Dyer extraction method^30,57^ was used to extract the lipid, which was then normalized by adipose tissue weight. Tissue samples of 300 mg were vortexed with 300 µL chloroform and 600 µL methanol five times, periodically after every minute. Then, 300 µL chloroform was added, and the mixture was homogenized for five minutes (1:1, v/v chloroform/methanol ratio) on ice. Next, 300 µL double-distilled water was added and the mixture filtered using a 40 µm filter. The filtrate was incubated on ice for 10 min, and then centrifuged at 2000 rpm for 5 min at 4 °C. The lower organic phase containing chloroform was transferred to a new glass vial. The lipid from the aqueous layer and the leftover adipose tissue from the filter were re-extracted with 500 µL of 1:1 chloroform/methanol (v/v). The chloroform layers were pooled and dried with a nitrogen stream. The lipid content was weighted and normalized using total tissue weight = Lipid extracted (mg)/adipose tissue weight (mg). The lipid content of the injected (right side) vs. non-injected control (left side) subcutaneous adipose tissue (AT) was calculated using the following equation.

$$\% {{{\rm{Lipid}}}}\; {{{\rm{Content}}}}\; {{{\rm{Change}}}}({{{\rm{mg}}}}/{{{\rm{mg}}}})=\\ \frac{{{{\rm{Lipid}}}}\; {{{\rm{in}}}}\; {{{\rm{Right}}}}\; {{{\rm{AT}}}}-{{{\rm{Lipid}}}}\; {{{\rm{in}}}}\; {{{\rm{Left}}}}\; {{{\rm{AT}}}}}{{{{\rm{Lipid}}}}\; {{{\rm{in}}}}\; {{{\rm{Left}}}}\; {{{\rm{AT}}}}}\times 100$$

(Right AT, H_2_S gel/CD injected side; Left AT, non-injected control side.)

The aqueous upper layer of tissue homogenates was centrifuged again at 2000 rpm for 5 min at 4 °C, and supernatant was collected for western blot analysis.

### Adipocyte size and lipid droplet size measurement

Adipose tissues were first collected and stored at −80 °C until processing. Prior to imaging and staining, tissues were cryosectioned into 5 µm thick sections and mounted on adhesive-coated Superfrost Plus slides. The sections were then thawed at room temperature for 10 min, after which images of unstained sections were captured using an IX71 Inverted Microscope (Olympus) for later analysis. These images were stored for subsequent processing in ImageJ. Immediately after imaging, the tissue sections were fixed in 4% paraformaldehyde (PFA) at 4 °C for one hour, followed by three PBS washes (5 min each) to remove residual fixative.

#### Cell size analysis

Stored images of unstained sections were processed in ImageJ by converting them to grayscale. To enhance contrast, the “Enhance Contrast” function was applied, normalizing brightness and setting saturated pixels to 0.3%. If the images exhibited uneven illumination, the rectangular selection tool was used to divide the image into quadrants for separate analysis. Thresholding was applied to distinguish cells from the background, with adjustments to the red-highlighted regions for optimized segmentation. The images were then converted to binary, and morphological processing, including hole filling, erosion, and dilation, was applied to refine cell structures. To separate overlapping cells, the Watershed function was used, ensuring clear boundary definition. Cell size was measured using the Analyze Particles function, with appropriate size and circularity filters. When necessary, pixel-based measurements were converted to micrometer units using a known scale for accurate quantification.

#### Perilipin and Oil red O immunofluorescence

Following imaging of unstained sections, immunofluorescence staining was performed to visualize macrophages and adipocyte membranes. After fixation, sections were blocked with 5% bovine serum albumin (BSA) in PBST (PBS with 0.1% Tween-20) for one hour to prevent non-specific binding. Primary antibody incubation was performed using Perilipin (Cell Signaling Technology, Danvers, MA, USA) at a 1:100 dilution in 5% BSA in PBST, followed by another PBS rinse. Secondary antibody incubation was conducted using Goat anti-Rabbit IgG (H + L) (Thermo Fisher, Waltham, MA, USA) at a 1:00 dilution in 5% BSA in PBST for 1 h.

For lipid accumulation assessment, Oil Red O staining was performed after immunofluorescence imaging. Sections were incubated in Oil Red O solution (saturated in 70% ethanol) for 15 min, followed by gentle rinsing. Finally, the stained sections were mounted in 10% glycerol in PBS with DAPI and covered with a coverslip. Sections were visualized under a fluorescence microscope, where Perilipin-labeled adipocyte lipid droplet (green), Oil red O (red) and nuclei (blue) were identified. To maintain fluorescence signal integrity, all procedures were performed in the dark, and slides were protected from light exposure. Lipid droplet morphology was analyzed using DP2-BSW Version 2.1q (Olympus), and droplet diameters were measured using ImageJ software (National Institutes of Health, USA.

### Immunofluorescence detection of macrophages in adipocyte cryosections

Macrophage detection in adipocyte cryosections was performed using immunofluorescence staining with an anti-CD68 antibody, while Caveolin was used as a cell membrane marker to delineate adipocyte boundaries. Adipose tissue cryosections (5–10 µm thickness) were mounted on adhesive-coated slides (Superfrost Plus) and thawed at room temperature for 10–15 min. The sections were fixed in 4% PFA for 1 h at 4 °C, followed by three PBS washes (5 min each) to remove residual fixative. To minimize non-specific binding, sections were incubated with a blocking buffer containing 5% BSA in PBST for 1 h at room temperature in a humidified chamber.

Primary antibody incubation was performed using CD68 (1:100 dilution; Cell Signaling Technology, Danvers, MA, USA) and Caveolin (1:100 dilution), both diluted in 5% BSA in PBST. The slides were incubated overnight at 4 °C in a humidified chamber to enhance specificity. Following incubation, the slides were washed three times with PBS (5 min each). For Immunofluorescence, the secondary antibodies, Alexa Fluor 594 Goat anti-Mouse IgG (H + L) (1:200 dilution) for CD68; Alexa Fluor 488 Goat anti-Rabbit IgG (H + L) (1:200 dilution) for Caveolin, and, for CD68 immunohistochemistry, Peroxidase-linked Goat anti-Mouse antibody (Calbiochem) (1:200 dilution) were diluted in 5% BSA in PBST and applied to the sections. The slides were incubated for 1 h at room temperature in a humidified chamber, followed by three PBS washes (5 min each) to remove excess antibody.

For nuclear visualization in immunofluorescence, sections were counterstained with 10% glycerol in PBS with DAPI (1 µg/mL in PBS) and covered with a coverslip. The stained sections were examined using a fluorescence microscope, where CD68-positive macrophages were identified based on their Alexa Fluor 594 (red) fluorescence. Caveolin-labeled cell membranes were visualized with Alexa Fluor 488 (green), and DAPI-stained nuclei were detected under the DAPI filter (blue). To prevent dehydration, slides were kept hydrated throughout the procedure, particularly during washing and incubation steps, using a humidified chamber. Tissue detachment was minimized by using adhesive-coated slides (Superfrost Plus) and performing gentle washes. Background staining was reduced by optimizing blocking conditions and antibody dilutions.

**a. Macrophage detection in adipose tissue cryosections** was performed using ImageJ by isolating CD68-positive cells through color thresholding and particle analysis. The fluorescence image was first loaded into ImageJ and split into separate channels, with the red channel selected for analysis. To enhance specificity, a color threshold was applied, adjusting the hue, saturation, and brightness to highlight CD68+ macrophages while minimizing background fluorescence.

Following thresholding, the binary mask was refined using morphological operations to remove noise and separate overlapping macrophages. Watershed segmentation was used for further object separation. To quantify macrophages, particle analysis was performed, setting an appropriate size threshold (10–Infinity µm^2^) and circularity filter (0.4–1.0) to exclude artifacts. The number, area, and distribution of CD68+ cells were recorded, and an overlay was generated to visualize detected macrophages on the original image.

**b. To quantify cell size in adipose tissue sections** stained with fluorescence markers, ImageJ was used for image processing, segmentation, and measurement. The fluorescence image was first loaded into ImageJ, and the green fluorescence signal, corresponding to cell boundaries, was isolated followed by selecting the Caveolin-1 green (S) channel. Using the freehand selection tool, individual adipocyte boundaries were manually traced and measured. For each cell, parameters such as area, perimeter, and Feret’s diameter were recorded. Additionally, lipid droplet size was quantified using Oil Red O and Perilipin co-stained sections, allowing for a detailed, cell-by-cell analysis.

Next, a color thresholding method was applied to extract the green-stained regions representing adipocyte membranes. The Hue (H) range was set between 35–90, and the Saturation (S) and Brightness (B) levels were adjusted to eliminate background noise and highlight cell structures. After applying the threshold, the resulting binary mask was refined using morphological operations. Specifically, dilation and erosion were performed to smooth the segmented regions, followed by hole filling to ensure complete cell detection. To further separate overlapping cells, the watershed transformation was applied.

For accurate size measurements, the scale bar present in the image was used to set the pixel-to-micrometer ratio. Cell size quantification was conducted through particle analysis, with size thresholds set to 10-Infinity µm^2^ to exclude artifacts and non-cellular components. The circularity range was adjusted to 0.3–1.0 to focus on adipocyte structures while excluding elongated artifacts. The analysis provided measurements including cell area (µm^2^), perimeter, and equivalent diameter.

### Picrosirius Red staining for fibrosis in adipose tissue

Adipose tissue cryosections were prepared and stained with Picrosirius Red to assess collagen deposition and fibrosis. Fresh-frozen adipose tissue was sectioned at 5 µm thickness and mounted onto glass slides. The sections were fixed overnight in 4% PFA at 4 °C, followed by three PBS washes (5 min each) to remove residual fixative. After fixation, sections were briefly rinsed in distilled water before proceeding to staining.

To enhance collagen visualization, the sections were incubated in 0.2% phosphomolybdic acid for 5–10 min to remove non-collagenous components. The sections were then stained with 0.1% Picrosirius Red (Direct Red 80 in saturated picric acid) for 60 min at room temperature, ensuring full immersion in the staining solution. After staining, excess dye was removed with a brief rinse in 0.01 N hydrochloric acid (HCl).

For optimal visualization, slides were rinsed in acidified water (0.5% acetic acid in distilled water) and mounted using an aqueous-based or resinous mounting medium. Coverslips were applied carefully to prevent air bubbles. Stained sections were examined under polarized light microscopy, where collagen fibers appeared red or orange, indicating fibrosis, while non-collagenous components appeared yellow or green. The intensity and distribution of Picrosirius Red staining were analyzed to assess the degree of adipose tissue fibrosis.

### Western blotting

The aqueous layer of tissue homogenates containing protein was treated with a lipid removal agent (Sigma-Aldrich, St. Louis, MO, USA) and placed on a rocker/orbital shaker at 4 °C for 30 min. Samples were then centrifuged at 15,000 rpm for 15 min, and the supernatant was collected for further analysis. Equal amounts of protein (20 μg in 20 μL/well) were denatured and resolved by SDS-PAGE, then transferred onto nitrocellulose membranes (Pall Corporation, Pensacola, FL). Membranes were blocked in 5% milk in TBST and incubated overnight at 4 °C on a shaker with primary antibodies (1:1000 dilution unless otherwise stated). Following washing, membranes were incubated with HRP-conjugated secondary antibodies (1:2000) and developed using enhanced chemiluminescence (GE Healthcare, Amersham, UK).

The following primary antibodies were used:

1. Hormone-Sensitive Lipase (HSL)—Mouse monoclonal antibody, Santa Cruz Biotechnology, sc-74489 (1:1000)
2. Phospho-HSL (Ser563)—Rabbit polyclonal antibody, Cell Signaling Technology, #4139 T (1:1000)
3. Adiponectin (C45B10)—Rabbit monoclonal antibody, Cell Signaling Technology, #2789, Lot 2 (1:1000)
4. PPARγ (C26H12)—Rabbit monoclonal antibody, Cell Signaling Technology, #2435 (1:1000)
5. SREBP-1 (E9F4O)—Rabbit monoclonal antibody, Cell Signaling Technology, #95879 (1:1000)
6. Perilipin 1 (K117)—Rabbit monoclonal antibody, Cell Signaling Technology, #3467S (1:1000 for WB, 1:100 for Immunofluorescence(IF))
7. α-Tubulin—Rabbit monoclonal antibody, Cell Signaling Technology, #2144S (1:1000)
8. β-Actin—Mouse monoclonal antibody, Sigma-Aldrich, #A2228 (1:1000)
9. CD68—Mouse monoclonal antibody, Invitrogen, #2923549 (1:100 for IF)
10. Caveolin-1—Rabbit monoclonal antibody, Cell Signaling Technology, #3238 (1:400 for IF)

Secondary antibodies compatible with rabbit and mouse IgG were used as appropriate for Western blotting and immunofluorescence.

### Cell culture to observe the role of H_2_S in SREBP activation

After primary preadipocytes were cultured and differentiated into mature adipocytes over a period of seven days^12^, treatments were administered. It was essential to ensure that terminal differentiation was complete before beginning any further analyses. Adipocytes were divided into four treatment groups to assess the effects of SREBP inhibition and H_2_S exposure during differentiation:

1. Control Group: Adipocytes were differentiated under standard conditions without any additional treatment, serving as a negative control^12^.
2. H_2_S Differentiation Group: 60 µM NaHS was added throughout differentiation (days 0–7) to examine the effects of H_2_S exposure on adipogenesis.
3. Differentiation Inhibition Group: 10 µM betulin was added throughout the differentiation period (days 0–7) to assess the effect of early SREBP inhibition on adipogenesis.
4. Differentiation + H_2_S Group: 10 µM betulin was added throughout differentiation (days 0–7), and 60 µM NaHS was added during differentiation (days 0–7) to determine whether H_2_S can modulate the effects of early SREBP inhibition.

After the treatment period, adipocytes Oil Red O staining was conducted to quantify triglyceride accumulation, providing a functional measure of lipid storage^12^.

### Statistical analysis and reproducibility

All data were analyzed using Microsoft Excel Version 2403 (Redmond, WA, USA) and GraphPad Prism 10.2.1 software (La Jolla, CA, USA), with results expressed as mean ± standard error of the mean (SEM). Criteria for exclusion were established beforehand, excluding extreme outliers beyond the 99% confidence interval of the mean and those exceeding three times the SEM; however, no outliers were identified. Significance was determined at *p* < 0.05. Sample sizes (*n* = 6 per group) were chosen based on prior studies showing consistent effects, ensuring statistical reliability while balancing ethical and cost considerations. Animal experiments utilized six independent replicates, while the HPLC method employed five replicates. Unpaired two-tailed Student’s *t*-tests were employed for comparisons between two groups, as appropriate. For analyses involving three or more groups, either one-way or two-way analysis of variance (ANOVA) was conducted, followed by Tukey’s multiple comparison post hoc test. In animal studies, sample sizes were determined to balance statistical power with cost and ethical considerations for animal welfare. Random allocation of samples/animals into experimental groups was performed at the study’s outset.

To ensure reproducibility, all experimental protocols—including sample preparation, data acquisition, and statistical analysis follow standardized operating procedures.

### Reporting summary

Further information on research design is available in the Nature Portfolio Reporting Summary linked to this article.

## Supplementary information

Supplementary Information

Description of Additional Supplementary Materials

Supplementary Data 1

Reporting Summary

## Data Availability

Supplementary information file contains uncropped and unedited blot/gel images. The source data for the graphs presented in this study are provided as an Excel file in Supplementary Data 1. Any additional data or supporting information will be made available from the corresponding author upon reasonable request.
