# Supplementary material for: Localized delivery and retention of hydrogen sulfide causing regional lipid accumulation in mouse adipose tissues in vivo
Source: Commun Biol. 2025 Jul 1;8:963. doi: 10.1038/s42003-025-08353-9 (PMC12216921; doi:10.1038/s42003-025-08353-9)
Supplement: Supplementary file 1 — Supplementary Information [file 42003_2025_8353_MOESM1_ESM.pdf]

Localized delivery and retention of hydrogen sulfide causing regional lipid accumulation in mouse adipose tissues *in vivo*

Richa Verma[1], Ming Fu[2,3], Hassan M. Arif [3], Guangdong Yang[1], Kuljeet Kaur,[4] John B. Matson[4], Lingyun Wu[1,3 *], Rui Wang[2,3*]

[1] Cardiovascular and Metabolic Research Unit, Laurentian University, Sudbury, Canada

[2] College of Basic Medicine, Shandong Second Medical University, Weifang, China

[3] Department of Biology, York University, Toronto, Canada

[4] Department of Chemistry, Macromolecules Innovation Institute, and Virginia Tech Centre for Drug Discovery, Blacksburg, USA

Raw uncropped images of all Western blots

Figure 3b

CSE

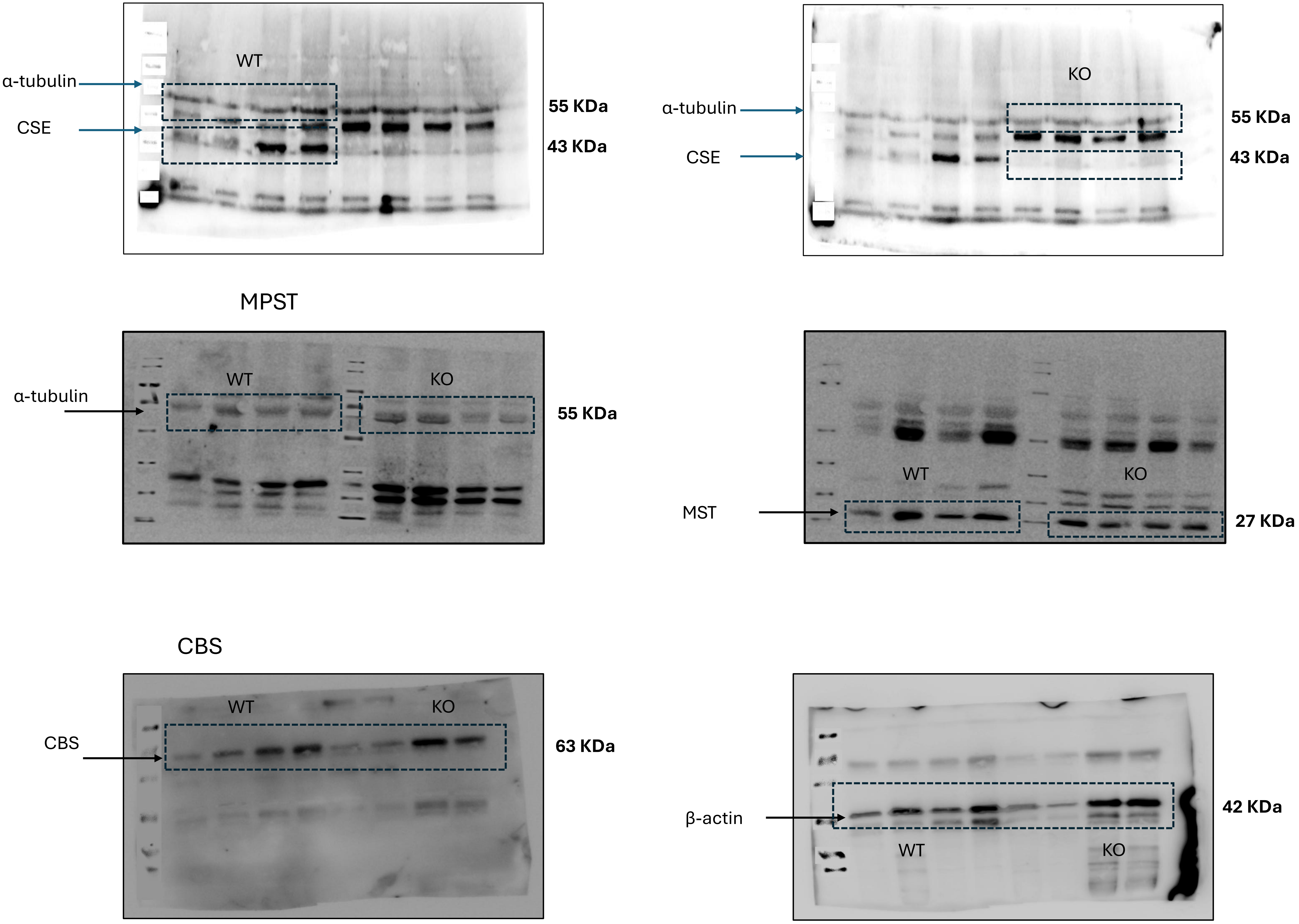

Figure 5a

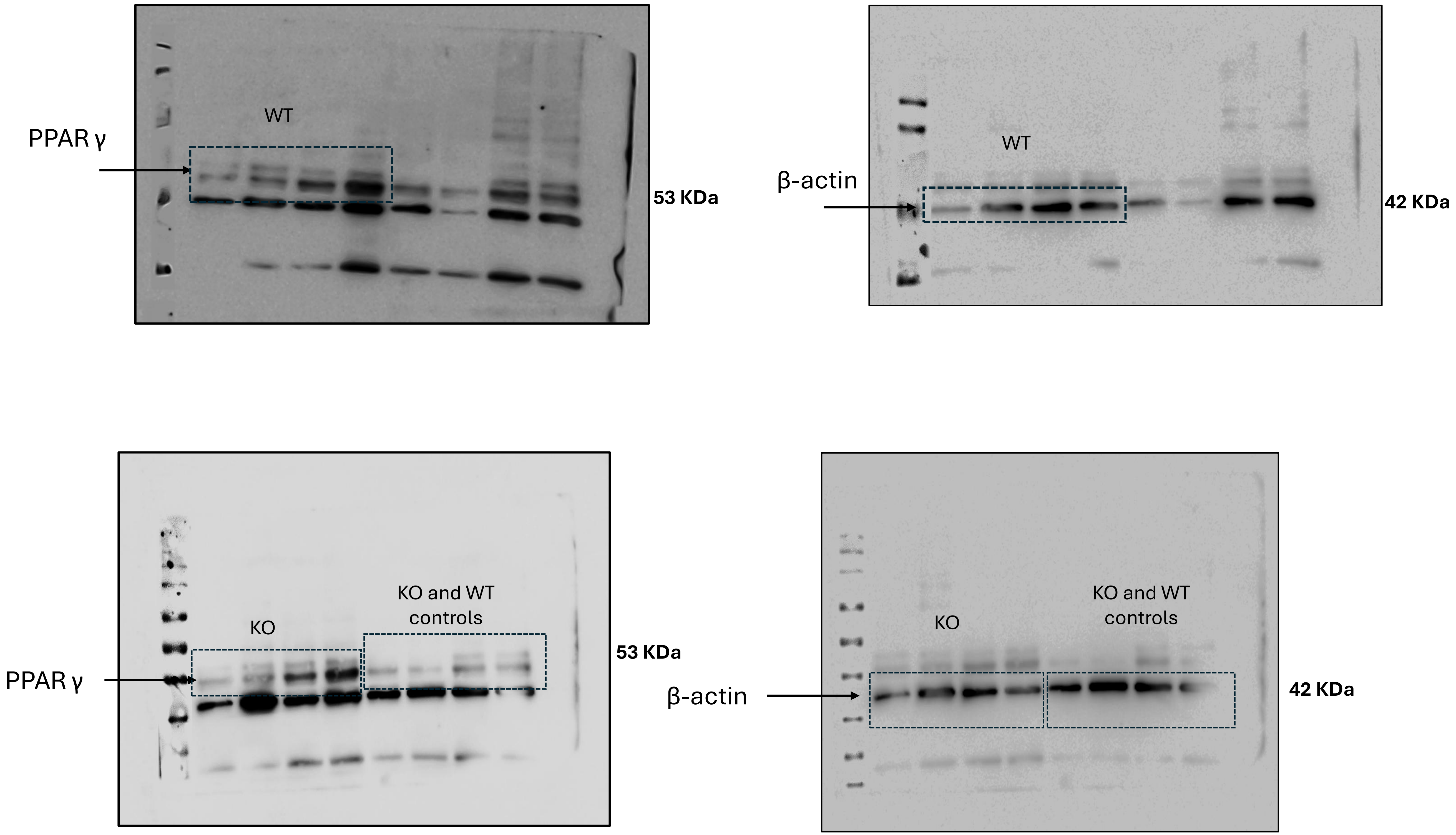

PPAR γ
WT
53 KDa
β-actin
WT
42 KDa
PPAR γ
KO
KO and WT controls
53 KDa
β-actin
KO
KO and WT controls
42 KDa

Figure 5a

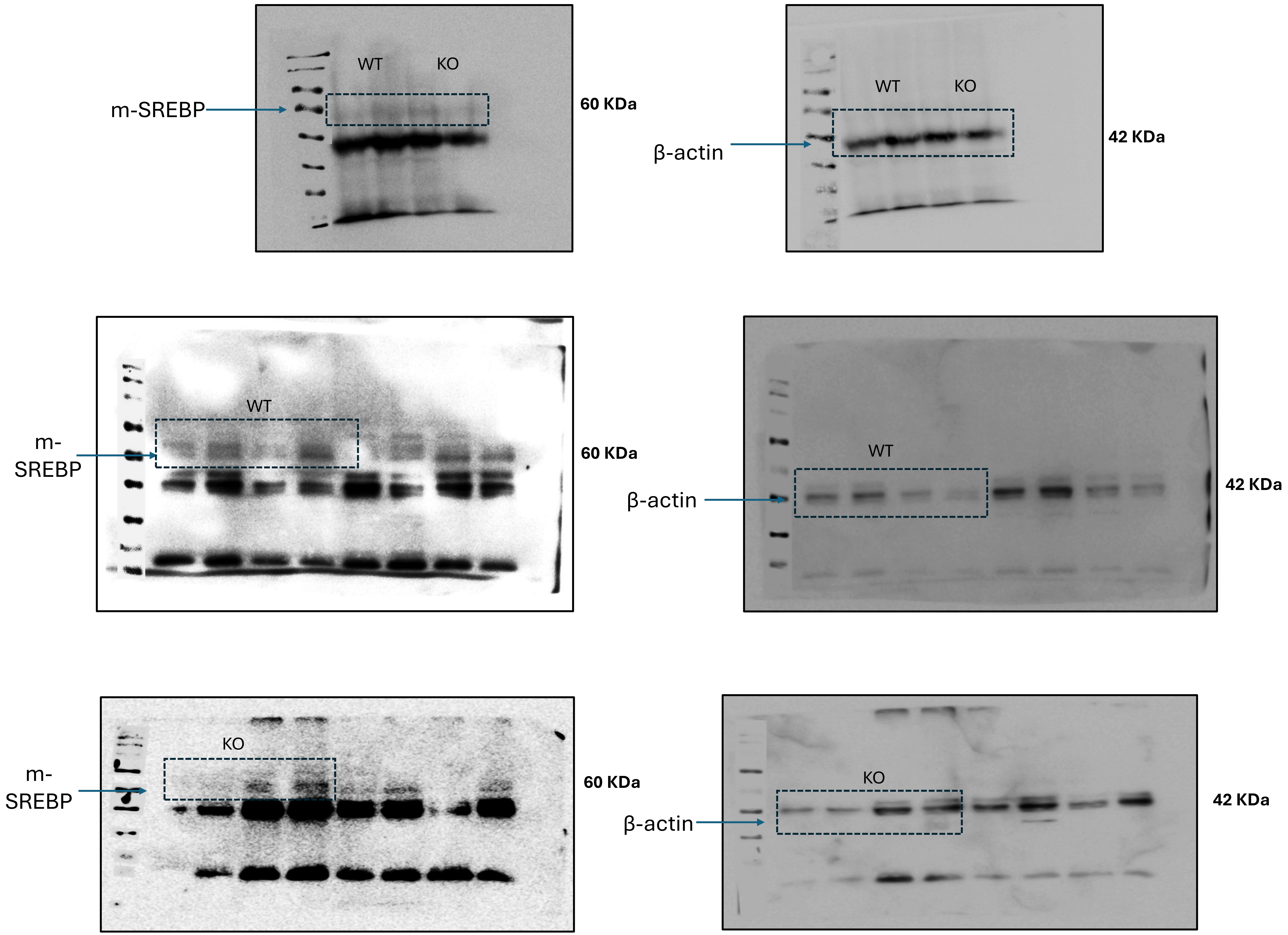
WT
KO
m-SREBP
60 KDa
WT
KO
β-actin
42 KDa
WT
m-SREBP
60 KDa
WT
β-actin
42 KDa
KO
m-SREBP
60 KDa
KO
β-actin
42 KDa

Figure 5a

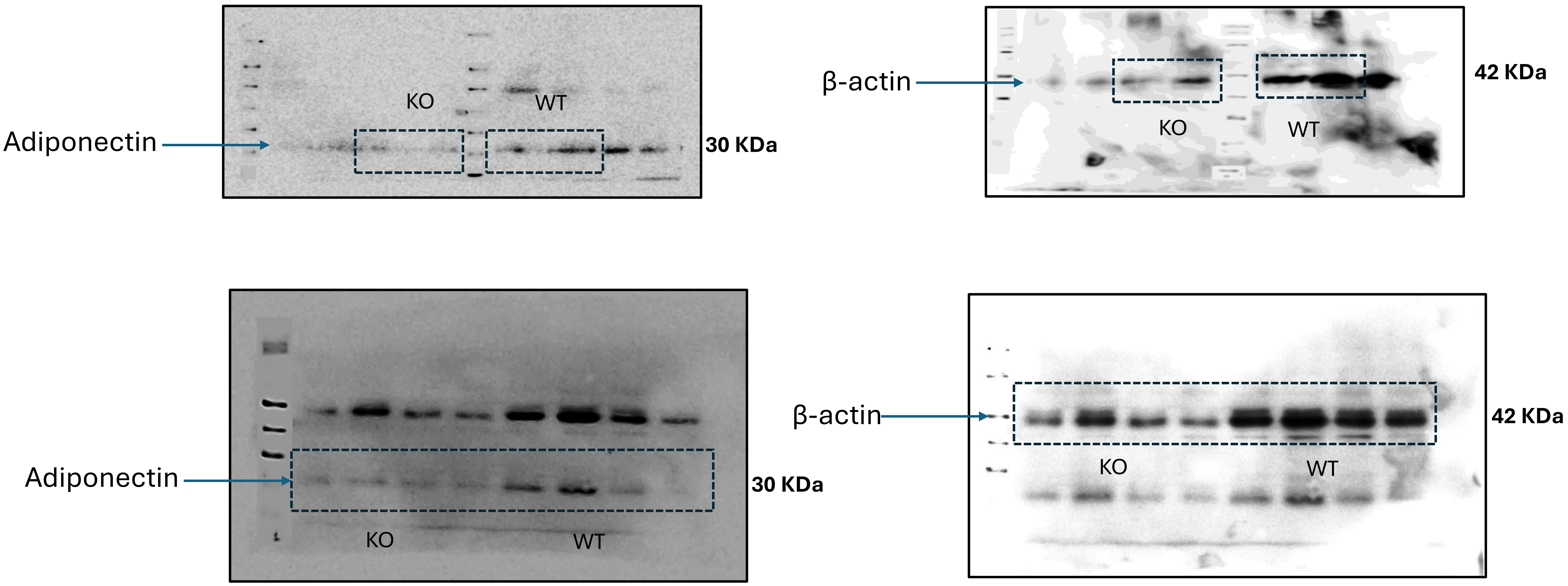

Figure 7a

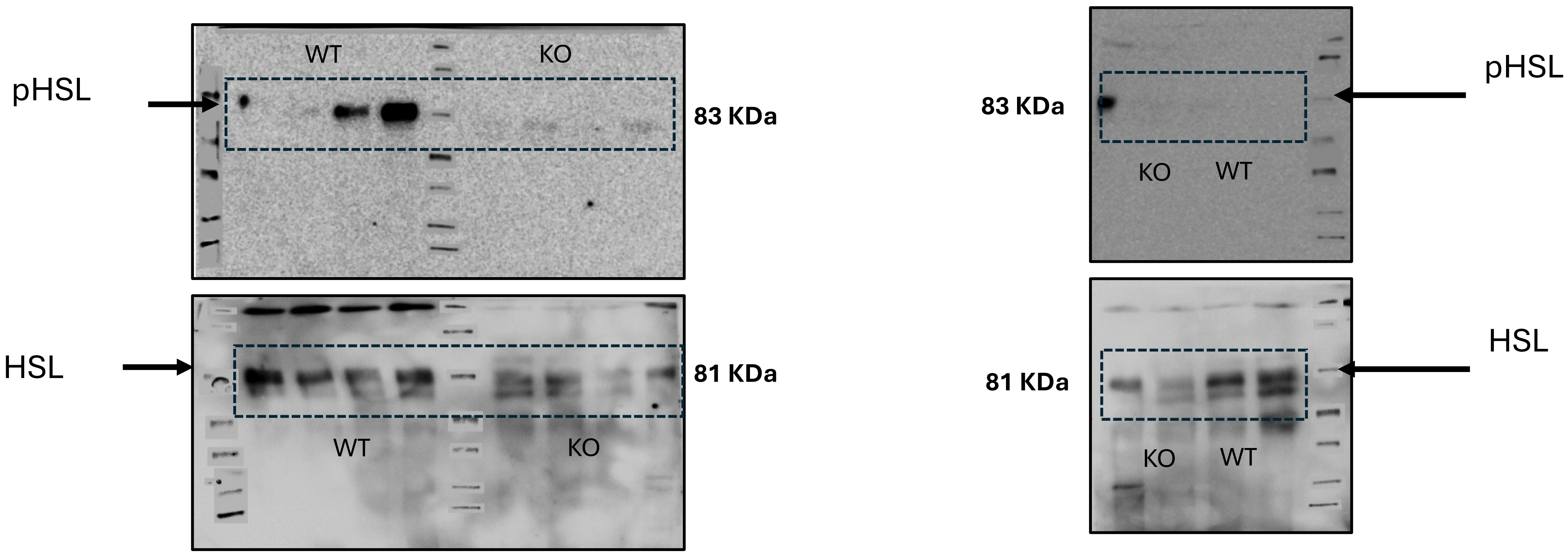

Figure 7a

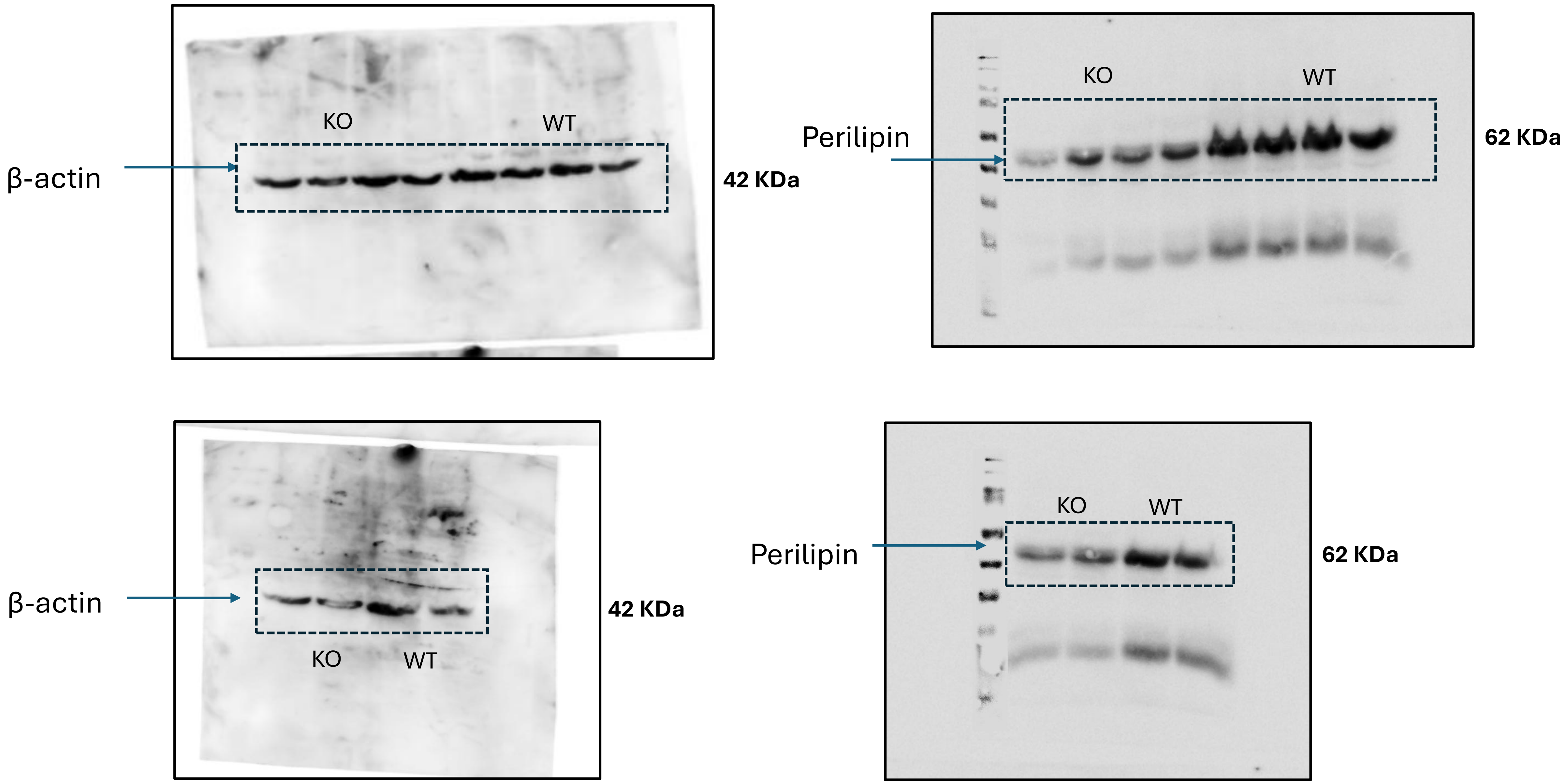

KO
WT
β-actin
42 KDa
KO
WT
Perilipin
62 KDa
β-actin
KO
WT
42 KDa
KO
WT
Perilipin
62 KDa
