# Supplementary material for: Localized delivery and retention of hydrogen sulfide causing regional lipid accumulation in mouse adipose tissues in vivo
Source: Commun Biol. 2025 Jul 1;8:963. doi: 10.1038/s42003-025-08353-9 (PMC12216921; doi:10.1038/s42003-025-08353-9)
Supplement: Supplementary file 2 — Description of Additional Supplementary Materials [file 42003_2025_8353_MOESM2_ESM.pdf]

Description of Additional Supplementary Files

File name: Supplementary Data 1

Description: The source data for the graphs presented in this study
